# Get+Connected: Development and Pilot Testing of an Intervention to Improve Computer and Internet Attitudes and Internet Use Among Women Living With HIV

**DOI:** 10.2196/resprot.6391

**Published:** 2017-03-31

**Authors:** Gabriela Seplovich, Keith J Horvath, Lorlette J Haughton, Oni J Blackstock

**Affiliations:** ^1^ Mailman School of Public Health Columbia University New York, NY United States; ^2^ Division of Epidemiology & Community Health University of Minnesota Minneapolis, MN United States; ^3^ Montefiore Medical Center/Albert Einstein College of Medicine Division of General Internal Medicine Bronx, NY United States

**Keywords:** women and HIV, Internet literacy, technology intervention, digital divide, information seeking behavior, access to health information

## Abstract

**Background:**

For persons living with chronic medical conditions, the Internet can be a powerful tool for health promotion, and allow for immediate access to medical information and social support. However, women living with human immunodeficiency virus (HIV) in the United States face numerous barriers to computer and Internet use. Health behavior change models suggest that the first step towards adopting a new health behavior is to improve attitudes towards that behavior.

**Objective:**

To develop and pilot test Get+Connected, an intervention to improve computer and Internet attitudes and Internet use among women living with HIV.

**Methods:**

To develop Get+Connected, we reviewed the extant literature, adapted an existing curriculum, and conducted a focus group with HIV-positive women (n=20) at a community-based organization in the Bronx, New York. Get+Connected was comprised of five weekly sessions covering the following topics: basic computer knowledge and skills, identifying reliable health-related websites, setting up and using email and Facebook accounts, and a final review session. We recruited 12 women to participate in pilot testing. At baseline, we collected data about participants’ sociodemographic information, clinical characteristics, and technology device ownership and use. At baseline, intervention completion, and three months postintervention, we collected data regarding attitudes towards computers and the Internet (Attitudes Towards Computers and the Internet Questionnaire [ATCIQ]; possible scores range from 5-50) as well as frequency of Internet use (composite measure). To examine changes in ATCIQ scores and Internet use over time, we used generalized estimating equations. We also collected qualitative data during intervention delivery.

**Results:**

Among women in our sample, the median age was 56 years (interquartile range=52-63). All participants were black/African American and/or Latina. Seven participants (7/12, 58%) had a high school diploma (or equivalent) or higher degree. Ten participants (10/12, 83%) reported owning a mobile phone, while only one (1/12, 8%) reported owning a computer or tablet. Only one participant (1/12, 8%) reported having ever used the Internet or email. Internet nonusers cited lack of computer/Internet knowledge (6/11, 54%) and lack of access to a computer or similar device (4/11, 36%) as the main barriers to use. Over time, we observed an improvement in attitudes towards computers and the Internet (ATCIQ scores: 33.5 at baseline, 35 at intervention completion, and 36 at three months postintervention; *P*=.008). No significant increase in Internet use was observed (*P*=.61). Qualitative findings indicated excitement and enthusiasm for the intervention.

**Conclusions:**

In our sample of urban, technology-inexperienced HIV-positive women, participation in Get+Connected was associated with an improvement in attitudes towards computers and the Internet, but not Internet use. Changing attitudes is the first step in many health behavior change models, indicating that with improved access to computer and Internet resources, frequency of Internet use may also have increased. Future studies should consider addressing issues of access to technology in conjunction with Get+Connected.

## Introduction

The Internet is a powerful tool for health promotion and disease management, and allows for immediate access to medical information and social support. According to a 2014 Pew Research Survey, 87% of Americans use the Internet [1]. Seventy-two percent of Internet users reported looking online for health-related information [2], with over 60% searching for information on specific disease conditions and roughly half searching for medical treatments [3,4]. Among those diagnosed with a chronic medical condition, nearly half used the Internet for advice or information on their illness [5]. Persons with chronic medical conditions are more likely than those without a chronic medical condition to track their health profile and health indicators online [6]. The Internet also provides access to social support by facilitating linkages among persons with chronic medical conditions via peer networks, online forums, and chat rooms [7].

Despite the Internet’s seeming ubiquity and the increasing popularity of leveraging the Internet for health promotion, significant disparities exist with respect to use [8]. For example, recent studies suggest that leveraging the Internet for health information and social support can improve health-seeking behaviors and outcomes in persons living with human immunodeficiency virus (HIV) [9-12]. More specifically, health-related Internet use among persons living with HIV is associated with greater medication adherence and use of active coping strategies, higher self-efficacy and CD4 white blood cell count, and an increased sense of social connectedness [13-15]. However, these studies were conducted with samples that were primarily male and white, and therefore may not be generalizable to other sociodemographic groups. Those who are economically disadvantaged, socially marginalized, and less educated are less likely to use the Internet for health-related purposes [16-18]. Among Internet users, including those living with HIV, there is a clear *digital divide*, with more affluent whites using the Internet more frequently than their African American and Latino counterparts, even after controlling for socioeconomic status [18-20]. The consequences of such disparities are profound and limit the potential resources that certain demographic groups living with HIV can leverage to improve their health.

Women living with HIV are largely socioeconomically disadvantaged, and therefore may be unlikely to reap the benefits of available electronic health information due to limited Internet access and/or use [21-25]. Among an urban, community-based sample of women living with HIV, Blackstock et al [26] found that the overall proportion of women using the Internet was lower than that of the general population (61% vs 84%). This study also found that reported barriers to Internet use among non-Internet-using HIV-positive women included a lack of interest, knowledge, and experience in navigating computers and the Internet [26]. However, among Internet-using women in the study, most (86%) were actively engaged in using the Internet for obtaining health-related and general information (75%), showing the potential benefits that could be reached once barriers to use are addressed [26].

According to the Theory of Planned Behavior, improving attitudes towards a behavior can enhance uptake of that behavior [27,28]. Therefore, among HIV-positive women, we sought to improve computer and Internet attitudes, and ultimately Internet use, by designing and pilot testing the *Get+Connected* intervention. This paper describes the development of *Get+Connected* as well as results from pilot testing of the intervention.

## Methods

### Get+Connected Intervention Development

The development of *Get+Connected* was based on a review of the literature on Internet use and health-related outcomes among HIV-positive women, adaptation of an existing online health information curriculum from the National Institutes of Health (NIH), and focus group findings [29,30]. The focus group was conducted at a community-based organization (CBO), which serves as a skilled nursing facility that provides onsite housing and supportive services to persons living with HIV in the Bronx, New York. Individuals were eligible to participate in the focus group if they self-identified as cis-gender women, were proficient in English, and expressed interest in participating in the focus group. Participants were actively recruited to participate by a CBO staff member who described the study in person to potential participants. Written informed consent was obtained from all participants. Traditionally, focus groups are conducted with a smaller number of participants; however, due to scheduling challenges, we conducted one focus group with 20 participants. Due to this known limitation, the focus group facilitators used strategies to help ensure that all participants were given equal time to respond to questions and accompanying probes; these included directly asking underrepresented participants their opinions, presenting multiple perspectives, reminding participants that there is no *correct answer*, and providing ample time for participants to organize and vocalize their thoughts. Participants were asked about their prior experiences with computers and the Internet, as well as what online skills they were most interested in learning (eg, *search for health-related information*, *use email*). Participants received US $10 and a round-trip Metrocard (value US $5) for their time. The study received Institutional Review Board (IRB) approval from Albert Einstein College of Medicine.

To identify prominent themes related to computer and Internet interest and use, we conducted a content analysis of the focus group transcript using a qualitative descriptive approach [31]. The focus group transcript was sorted into several content areas related to computers and the Internet, including experience, interest, and desired skills or related activities (eg, how to email, how to use social media). The transcripts were read several times by the study team. Text corresponding to these content areas were extracted and coded, and codes were consolidated into themes.

Our findings indicated that most women had minimal to no experience using a computer or the Internet. However, participants were interested in learning the following skills or activities: typing fluency and speed, how to set up an email account, and how to conduct an Internet search for general health-related purposes (eg, information on HIV, mental illness, managing chronic diseases and pain, general women’s health, and medication interactions). Participants expressed interest in: learning how to use social media sites, including Facebook, to reconnect with friends and family; joining support groups for persons living with HIV; and accessing online forums to ask questions and exchange information related to chronic disease management. The most commonly cited reasons for wanting to use the Internet were reconnecting with family and old friends, learning more about their health, and keeping abreast of technological trends (eg, many women expressed a desire to show their children and grandchildren that they could use email and Facebook). Similar to the findings of a previous study [26], lack of computer and/or Internet knowledge and access to technology were cited as additional barriers to Internet use.

### Intervention Description

*Get+Connected* consisted of five weekly 45-minute sessions held at the CBO. Each session was led by a member of the study team with one or two other members present to assist as needed. The curriculum (see Multimedia Appendices 1-4) was delivered using Microsoft PowerPoint presentations projected onto a screen at the front of the room. For each session, participants were provided with a laptop (Google Chromebook) to follow along with the lesson. If requested, a specialized ergonomically correct mouse was provided to participants with limited hand mobility. Focus group participants reported a lack of computer and/or Internet access, so all participants were given library cards and shown how to access the nearest public library (six blocks away from the CBO) as part of the intervention [32]. The library provides free computer and Internet use during business hours, in addition to other educational resources.

#### Session 1

The first session, which was delivered by a librarian from the local branch of the city library system who specializes in computer literacy for low-income individuals, introduced participants to the fundamentals of computer and Internet use. The lesson began with how to operate the laptop, including how to *wake up* the computer, how to use a mouse to navigate on the computer screen, and how to open an Internet browser. Participants received contextual information, including what a browser is and how browsers can be used to access information. Participants were asked to search for something basic online (eg, “cats”) and shown how to view online content, images, videos, and maps. Throughout this search, participants were shown how to scroll up and down on a web page, how to navigate forwards and backwards, how to copy and paste text, and how to save an image to the desktop. Participants also learned how to search for their local public library branch to access free computers and Internet service using Google Maps. Participants were reminded about the free computer access that was available, and were encouraged to visit the library whenever possible.

#### Session 2

The second session was intended to help participants identify reliable health websites based on defined criteria and was adapted from an NIH computer literacy training curriculum for seniors [30]. Modifications in language, format, and delivery were made to optimize the curriculum for the study’s target population. This lesson taught participants how to identify specific website components that gauge the reliability of a given site. These components include: identifying a website’s sponsor; identifying the purpose of the website; finding the site’s author, date of publication, or date of last update; and checking for plausibility of website content. Two websites were used as examples of trustworthy and credible sites for participants to identify the above components [33,34].

#### Session 3

This session sought to guide participants through setting up an email account, and taught them how to use email as a mode of communication. Participants created a new account using Gmail, were given basic tips on how to create a username and secure password, and were shown how to update their profiles. Instructions on how to compose a new email were provided, including: how to input an email address, subject heading, and text body; how to open incoming messages; and how to reply to messages. Participants with prior computer or Internet experience were taught to attach saved files and insert articles into their emails. Participants then practiced these skills by sending short emails to each other and to their friends and family.

#### Session 4

In session four, participants learned how to sign up for a Facebook account, but were first coached on safety and security issues. Following registration, participants were guided through Facebook’s features, including how to search for friends, how to send a friend request, how to accept or deny a friend request, how to comment on others’ Facebook pages, and how to browse through photos. Participants were also encouraged to *follow* relevant health websites, including *The Well Project*, a nonprofit organization dedicated to serving women and girls with HIV [35]. The instructor explained that such health websites periodically post relevant information, and guided participants on how to open posts of interest. Participants spent the remainder of the session independently navigating through their Facebook and email accounts.

#### Session 5

Session five was a review session incorporating all of the material learned in sessions 1 through 4. This session was intended to serve as a refresher course, and consisted primarily of the instructor asking individual participants to verbally and physically perform certain computer and Internet navigation functions. Participants were asked if they could: open a browser; conduct a Google search to find web content, images, and videos; and to find directions to their local library using Google Maps. Participants were then asked to reiterate the specific components of identifying a trustworthy website, open their email accounts to compose and reply to an email, and to navigate through their Facebook accounts to find health information of interest. The session concluded with participants receiving a certificate of completion for their efforts.

### Settings and Participants

*Get+Connected* pilot testing took place at the same CBO in which the focus group was conducted. Of the 20 women who participated in the focus group, 12 participated in the intervention. We conducted three waves of pilot testing with each cohort consisting of four participants. Written informed consent was obtained from all participants. The study received IRB approval from Albert Einstein College of Medicine.

### Data Collection

Quantitative data was collected from participants at baseline (immediately prior to session 1), intervention completion (immediately after session 5), and three months after completion of the intervention. Baseline self-report surveys were obtained using Audio Computer-Assisted Self-Interview (ACASI) technology on (1) sociodemographic and clinical characteristics, (2) technology device ownership and use, and (3) attitudes towards computers and the Internet along with frequency of Internet use. Follow-up surveys at intervention completion and at three months only collected data regarding attitudes towards computers and the Internet, and frequency of Internet use. During the study visits, two members of the study staff were available nearby to provide assistance with use of ACASI as needed. During the intervention sessions, we collected participants’ verbal feedback about *Get+Connected.*

### Measures

#### Sociodemographic and Clinical Characteristics

Sociodemographic characteristics included age, race (black/African American, white, Asian, Native Hawaiian or Pacific Islander, Native American or Alaskan Native, more than one race, or other), ethnicity (Hispanic or Latina), primary language (English, Spanish, French, Haitian Creole, Other, or don’t know), and highest grade completed in school. Clinical characteristics included self-perceived health status (excellent/very good/good vs fair/poor), comorbid medical conditions, and time since HIV diagnosis.

#### Intervention Retention

We calculated the average number of women in attendance at each session.

#### Technology Device Ownership and Use

We asked participants to report whether they owned the following devices: desktop, laptop or notebook, mobile phone, electronic book, or tablet. For those who owned a mobile phone, we asked whether they had used their mobile phones to do the following: send or receive texts, send or receive email, access the Internet, use apps to track health, or to look up health or medication information.

#### Attitudes Towards Computers and the Internet

At baseline, intervention completion, and three months after completion of the intervention, we used the Attitudes Towards Computers and the Internet Questionnaire (ATCIQ) to assess participants’ attitudes about computers and the Internet, which was adapted from the Attitudes Towards Computers Questionnaire [36] to include the Internet in addition to computers. The ATCIQ is comprised of 10 items, which include a 5-item computer/Internet interest subscale and a 5-item computer/Internet efficacy subscale. Responses for each item are ranked on a 5-point Likert scale ranging from *strongly disagree* (1) to *strongly agree* (5). Examples of questions include, “I know that if I worked hard to learn about computers/Internet, I could do well”, “Computers/Internet are *not* too complicated for me to understand” and, “I think I am the kind of person who would learn to use a computer/Internet well.” Potential total scores for the questionnaire range from 10 to 50, with a higher score indicating greater computer/Internet interest and/or efficacy. This tool has been recommended for use in socioeconomically disadvantaged demographics [18].

#### Internet Use

An *Internet user* was defined as an individual that had ever used the Internet or email (yes/no). At baseline, if the participant was an Internet user, they were asked about their Internet use over the past three months, including how many times they had used the Internet in general and for the following reasons: to email, to find general information, to search for health-related information, to search specifically for HIV-related information, to take part in chat rooms and online discussions with other people, to use social media networking sites, and to use Instagram. Participants’ responses to these items could include any number. At intervention completion and three months postintervention, participants were asked the same questions about their Internet use for the previous month. For each time point, we created a composite Internet use measure that included the sum of all individual Internet measures for each participant.

#### Statistical Analyses

We examined descriptive statistics for all variables at baseline. For attitudes towards computers and the Internet, we calculated median composite ATCIQ scores for the study sample at baseline, at completion of the intervention, and at three months postintervention. To examine whether there was a trend in composite ATCIQ scores over time, as well as the two ATCIQ subscales, we conducted analyses using generalized estimating equations (GEEs). Similarly, for the composite Internet use score, we calculated the median number of times the Internet or related technology was used at baseline and follow-up time points. To examine whether there was a trend of use in the composite measure of Internet use over time, we conducted analyses using GEEs. Due to our study’s limited sample size, adjustment for covariates was not preformed. All analyses were conducted using SAS statistical software (SAS 9.4, SAS Institute, Inc., Cary, North Carolina).

## Results

### Characteristics of Participants

We enrolled 12 participants in the pilot study. Median age was 56 years (interquartile range [IQR]=52-63). All but one participant self-identified as black or African American (11/12, 91%) and two self-identified as Latina ethnicity (2/12, 17%). English was the primary language for all participants. Seven participants had completed high school or higher degree (7/12, 58%), and nine rated their health as good, very good, or excellent (9/12, 75%). Half of the participants (6/12, 50%) reported having hypertension and 42% (5/12) reported a chronic lung disease. Median time since HIV diagnosis for our sample was 25.5 years (IQR=15-33).

### Retention in the Intervention

The average proportion of the study sample in attendance at each weekly session was 95% (range=75-100).

### Technology Device Ownership and Use

At baseline, no participants reported owning a desktop computer or e-book (Table 1). One participant reported owning a laptop computer or notebook and another reported owning a tablet. In contrast, 83% (10/12) of participants owned a mobile phone and approximately half of these individuals used it for texting. Use of mobile phones for other activities was minimal.

**Table 1:** Technology device ownership and use (n=12).

Measure	n (%)
Own a desktop computer	0/12 (0%)
Own a laptop computer or notebook	1/12 (8%)
Own a mobile phone	10/12 (83%)
Use mobile phone to send or receive texts	5/10 (50%)
Use mobile phone to send or receive email	1/10 (10%)
Use mobile phone to access the Internet	1/10 (10%)
Use mobile phone for apps to track your health	1/10 (10%)
Use mobile phone to look up health or medical information	1/10 (10%)
Own an electronic book or e-book reader	0/12 (0%)
Own a tablet computer	1/12 (8%)

### Attitudes Towards Computers and the Internet

There was a significant increase in composite ATCIQ scores over time. The median composite ATCIQ scores were 33.5 (IQR=30-34) at baseline, 35 (IQR=33-35) at intervention completion, and 36 (IQR=35-38) at three months postintervention (*P*=.008). For the ATCIQ computer/Internet interest subscale, there was also a significant increase over time (20, IQR=16.5-20.5 at baseline; 20, IQR=19-21 at intervention completion; and 23, IQR=20-25 at three months postintervention; *P*=.02). However, for the ATCIQ computer/Internet efficacy subscale, there was no significant change over time (13, IQR=11.5-14 at baseline; 14, IQR=13-15 at intervention completion; and 13, IQR=13-14 at three months postintervention; *P*=.40).

### Internet Use

At baseline, only one participant reported having ever used the Internet or email (1/12, 8%). Among the 11 participants who had never used email or the Internet (11/12, 92%), the main reasons for not using these technologies were not knowing how to email or use the Internet (6/11, 55%) and not having access to a computer or similar device (4/11, 36%). All participants reported that in order to start using email or the Internet they would need someone to help them. Only three participants had asked a friend or family member to look something up or complete a task on the Internet for them (3/11, 27%). We did not find a significant trend in our composite Internet use measure over time. Our median composite Internet use measure at baseline was 0 (IQR=0-0), 1 (IQR=0-20) at intervention completion, and 0 (IQR=0-11.5) at three months postintervention (*P*=.61).

### Participants’ Impressions of Get+Connected

Qualitative data showed that participants expressed excitement and approval for the intervention. Participants expressed enthusiasm for being able to connect with family members using social media and email (eg, *“Now I can talk with my grandkids more often!”; “My son was so happy when he saw my [first] email!* ”). Participants shared their eagerness to find old friends on social media (eg, *“I want to learn to use Facebook so I can find old friends.”*), and also expressed interest in being able to keep up with technological trends (eg, *“I want to be able to keep up with the times”; “I told my son I’m going to learn how to use Google Maps!”*). These findings allude to the acceptability of *Get+Connected* among our study sample.

## Discussion

### Principal Results

We developed and pilot tested *Get+Connected*, an intervention to improve computer and Internet attitudes and Internet use among women living with HIV. *Get+Connected* provided instructions on how to use a computer, navigate the Internet, conduct basic general and health-related Internet searches, and connect with others on a social media website. We also provided library cards for participants to access computers at their local library. We found that most women in our study owned mobile phones, but not other electronic devices. Mobile phones were used primarily for texting rather than Internet use. At baseline, only one participant reported ever using the Internet. The main reasons cited for lack of Internet use included lack of digital literacy and computer/Internet access. We found that participating in *Get+Connected* was associated with an improvement in women’s attitudes towards computers and the Internet over time. Specifically, interest in computers and the Internet increased over time; however, there was no significant change in computer and Internet efficacy. Additionally, Internet use did not increase over time.

There are several possible explanations for our findings regarding computer and Internet attitudes and Internet use. With regards to attitudes towards computers and the Internet, it is likely that exposure to computers and the Internet, and the instructions provided as part of *Get+Connected,* enabled participants to feel more comfortable and familiar with these technologies, thereby increasing computer and Internet interest. More computer and Internet use beyond that provided by the intervention may have been needed for participants to build greater confidence (ie, self-efficacy) in their ability to navigate computers and the Internet on their own. It is also conceivable that, due to living arrangements and limited physical mobility, providing information on how to use the Internet without increasing access to computers is insufficient to increase self-efficacy and actual Internet use. For example, many women had difficulty walking and the local library may not have been easily accessible to them. Additionally, the residential facility had only one working computer, and participants reported long wait times and restrictions on social media sites (including Facebook) which likely discouraged use. It is also possible that our small sample size may have precluded our ability to find a significant difference over time in our computer/Internet efficacy subscale and composite Internet use measure.

This pilot study of *Get+Connected* adds valuable information to the literature regarding computer and Internet use among individuals living with HIV. Previous research shows that the Internet can be a useful tool to improve the health of persons living with HIV. A study by Kalichman et al [12] demonstrated that an Internet skills training intervention for persons living with HIV improved Internet use for health, information, coping, and social support (compared to a control group). Another study found that increasing Internet health literacy for individuals living with HIV who have low literacy increases consumption of health information resources [24]. Unlike prior studies [37-40], our study focused specifically on HIV-positive women, a largely socioeconomically disadvantaged demographic group that faces unique health concerns and significant barriers to computer and Internet use. Therefore, this population may require specific interventions tailored to those needs. To our knowledge *Get+Connected* is the first intervention for persons living with HIV to include instructions in the use of social media that allows for peer-to-peer communication (ie, Web 2.0 technology). Social media, which can be leveraged to improve social support, has been shown to influence health outcomes among women with HIV [41,42].

### Areas for Future Research

Due to the ubiquity of mobile phones, future research should consider this platform for computer and Internet training interventions for HIV-positive women. Study participants had difficulty accessing a computer as well as the Internet, indicating that they were likely limited in their ability to practice what they had learned. Using mobile phones for Internet training may help to facilitate greater participation and practice outside of the classroom. Additionally, specific attention should be given to the types of mobile phone plans that participants have, in order to assess the feasibility of this approach. However, Internet access remains a significant barrier to use for women living with HIV. As such, future research will need to explore innovative approaches to providing Internet access to individuals with limited socioeconomic resources. Future interventions of this type should consider providing a list of locations with free WiFi access (eg, libraries, other CBOs). However, it is likely that more widespread policies will be needed to enhance Internet access, such as providing free WiFi in public spaces, funding Internet access at CBOs, and equipping low-incoming housing with free computers and WiFi. Unlike previous Internet skills training interventions for persons with HIV [24,37-39], our intervention provided instructions in the use of a social networking site (Facebook), a potentially important platform for harnessing social support and providing useful health information for women living with HIV. Future studies should consider how best to use social networking sites to connect people living with HIV with one another, and with informational resources.

### Limitations

There are important limitations to be considered in our study. First, our sample size was small and may have been underpowered to find an effect of the intervention on Internet use. However, it is important to note that this is a pilot study with the primary purpose of assessing the intervention’s feasibility and preliminary efficacy. Second, due to the nature of the study and limited resources, increasing Internet access was not addressed. Further studies should consider developing ways to increase Internet access, which remains a prominent barrier to Internet use in this demographic group. Third, while we found a significant difference in participants’ change in attitudes towards computers and the Internet over the follow-up period (as measured by the ATCIQ scale), there is no existing data on what would be considered a clinically meaningfully change in ATCIQ score; as such, we may need to be cautious in our interpretation of the intervention’s potential effect. Fourth, participants lived onsite at the CBO where the intervention took place and received weekly reminders to attend the *Get+Connected* sessions. Therefore, the high participation rate observed may not reflect the true acceptability of the intervention if participants had lived offsite from the intervention. Due to this limitation, our findings may not be generalizable to women with HIV who live in other settings. Lastly, our study did not include the evaluation of HIV-positive transgender women, which is a population that is also marginalized and lacks access to computer and Internet access. Future studies should consider the unique needs of this sociodemographic group and use the extant literature to develop interventions accordingly.

### Conclusions

Studies show that the Internet can be a powerful tool for health promotion, yet there is a clear divide in Internet use along socioeconomic lines. Among women living with HIV, *Get+Connected* sought to address this gap by improving attitudes towards and, ultimately, use of the Internet with this specialized training curriculum. Results showed that compared with baseline, participation in the intervention was associated with an improvement in women’s attitudes towards computers and the Internet over time. However, we did not find a change in participants’ Internet use over time. Further research focused on enhancing Internet access and use among women living with HIV should address issues of computer and Internet accessibility, and test other potential platforms (eg, mobile phones) for delivering similar interventions.

## Appendix

Multimedia Appendix 1

Multimedia Appendix 2

Multimedia Appendix 3

Multimedia Appendix 4

## Abbreviations

ACASI: Audio Computer-Assisted Self-Interview
ATCIQ: Attitudes Towards Computers and the Internet Questionnaire
CBO: community-based organization
GEE: generalized estimating equation
HIV: human immunodeficiency virus
IQR: interquartile range
IRB: Institutional Review Board
NIH: National Institutes of Health
