# Supplementary material for: Get+Connected: Development and Pilot Testing of an Intervention to Improve Computer and Internet Attitudes and Internet Use Among Women Living With HIV
Source: JMIR Res Protoc. 2017 Mar 31;6(3):e50. doi: 10.2196/resprot.6391 (PMC5392213; doi:10.2196/resprot.6391)
Supplement: Multimedia Appendix 1 [file resprot_v6i3e_app1.ppt]

## Slide 1
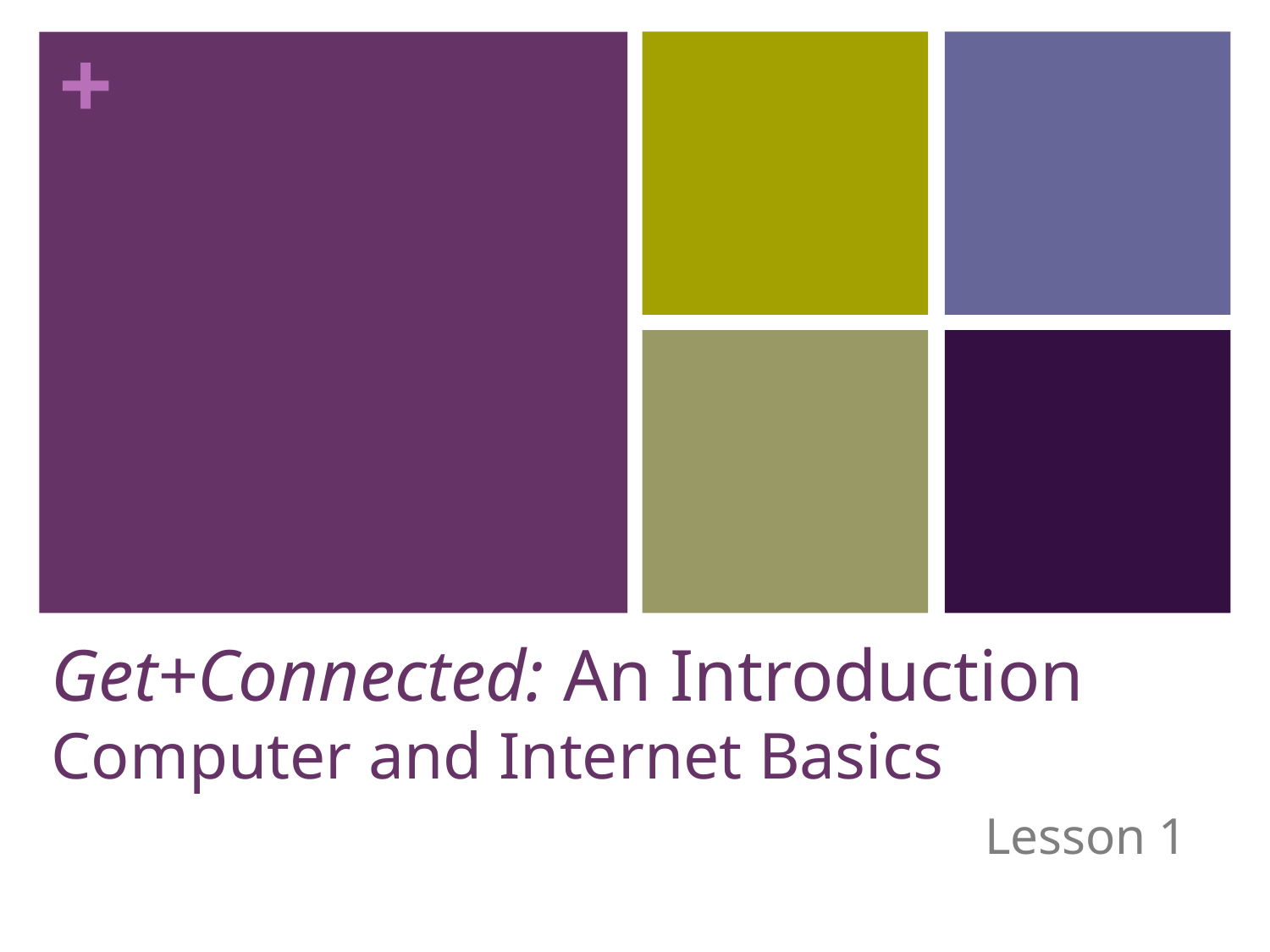

# Get+Connected: An Introduction Computer and Internet Basics
Lesson 1

## Slide 2
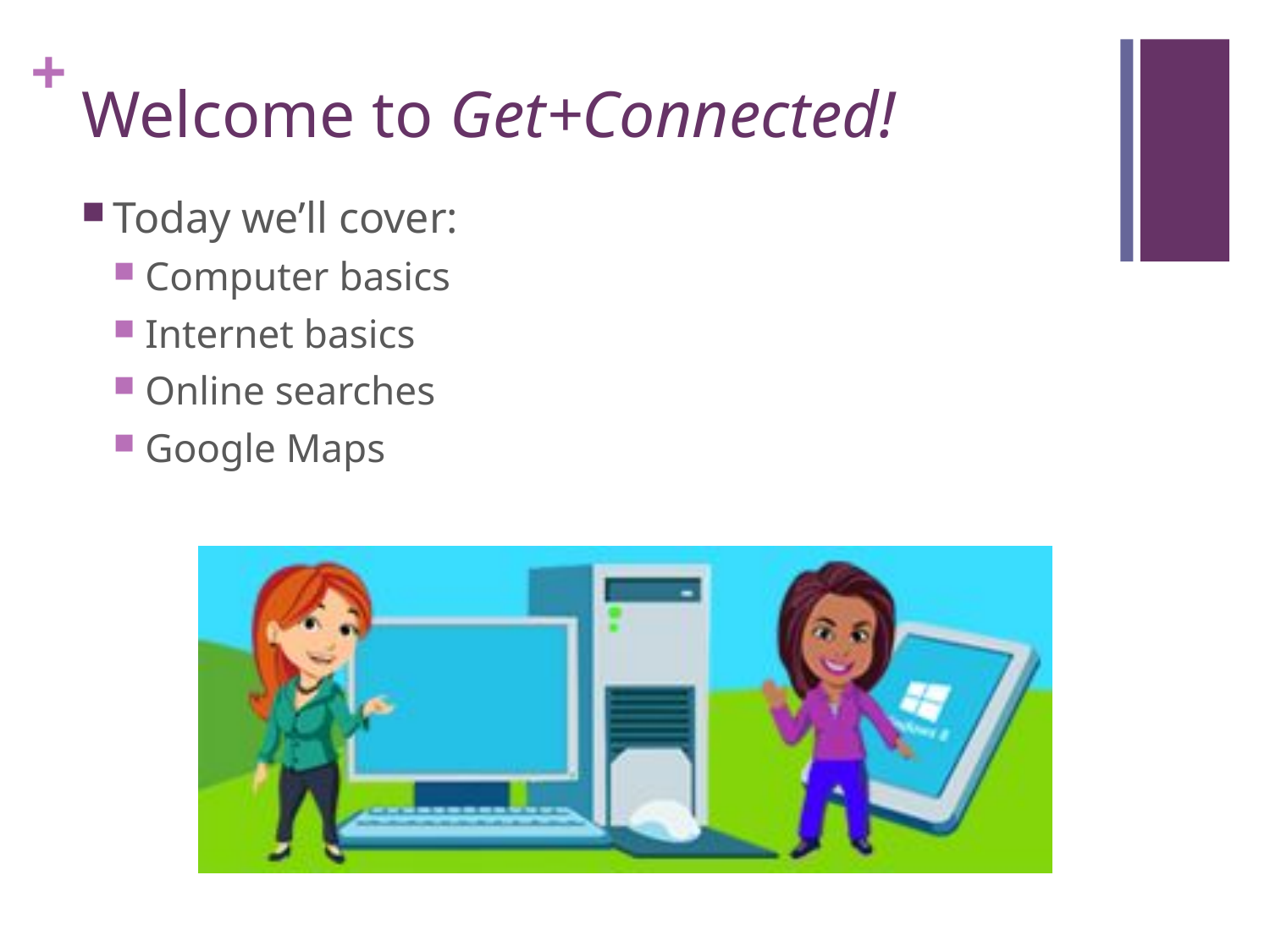

# Welcome to Get+Connected!
Today we’ll cover:
Computer basics
Internet basics
Online searches
Google Maps

## Slide 3
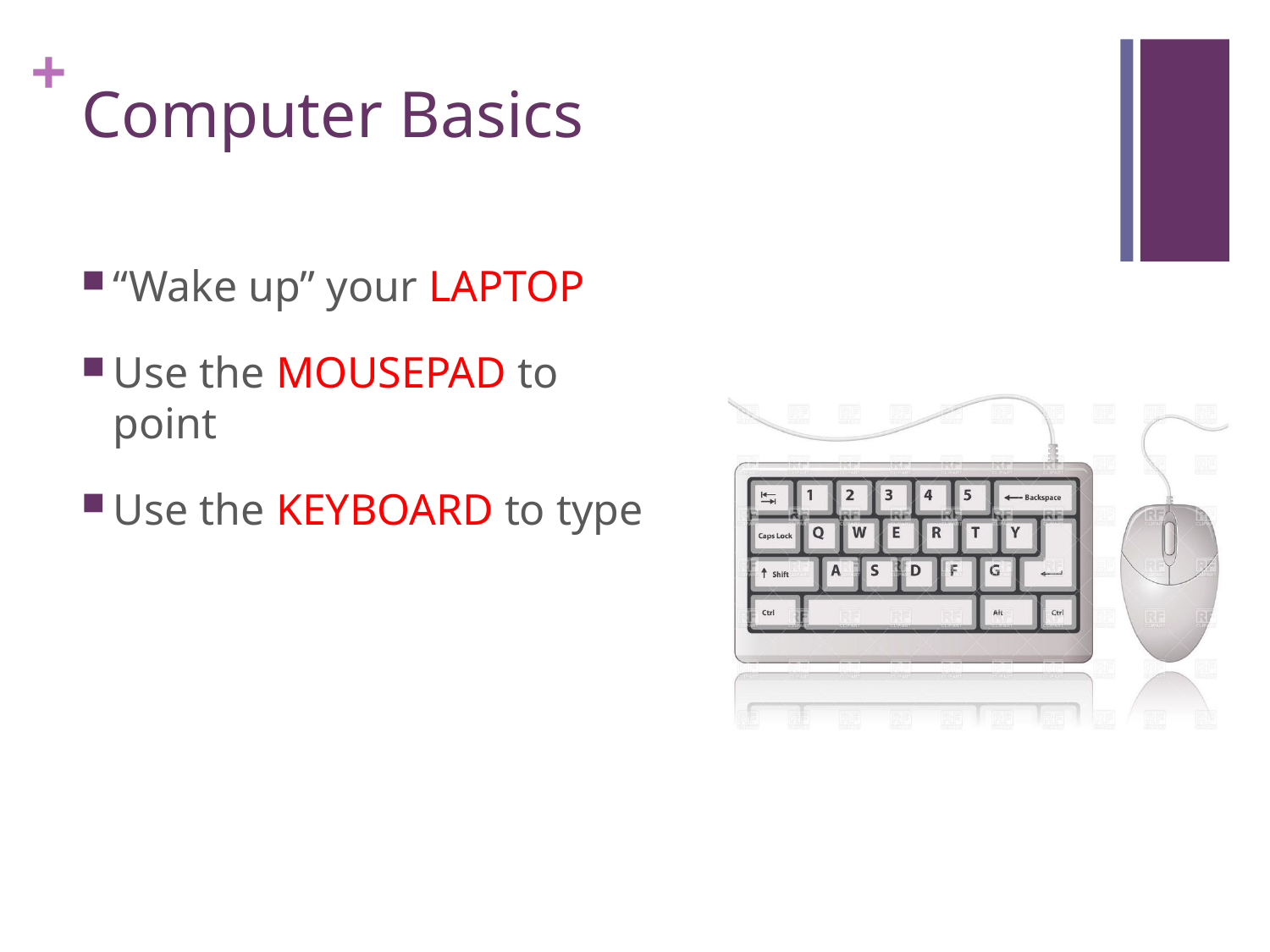

# Computer Basics
“Wake up” your LAPTOP
Use the MOUSEPAD to point
Use the KEYBOARD to type

## Slide 4
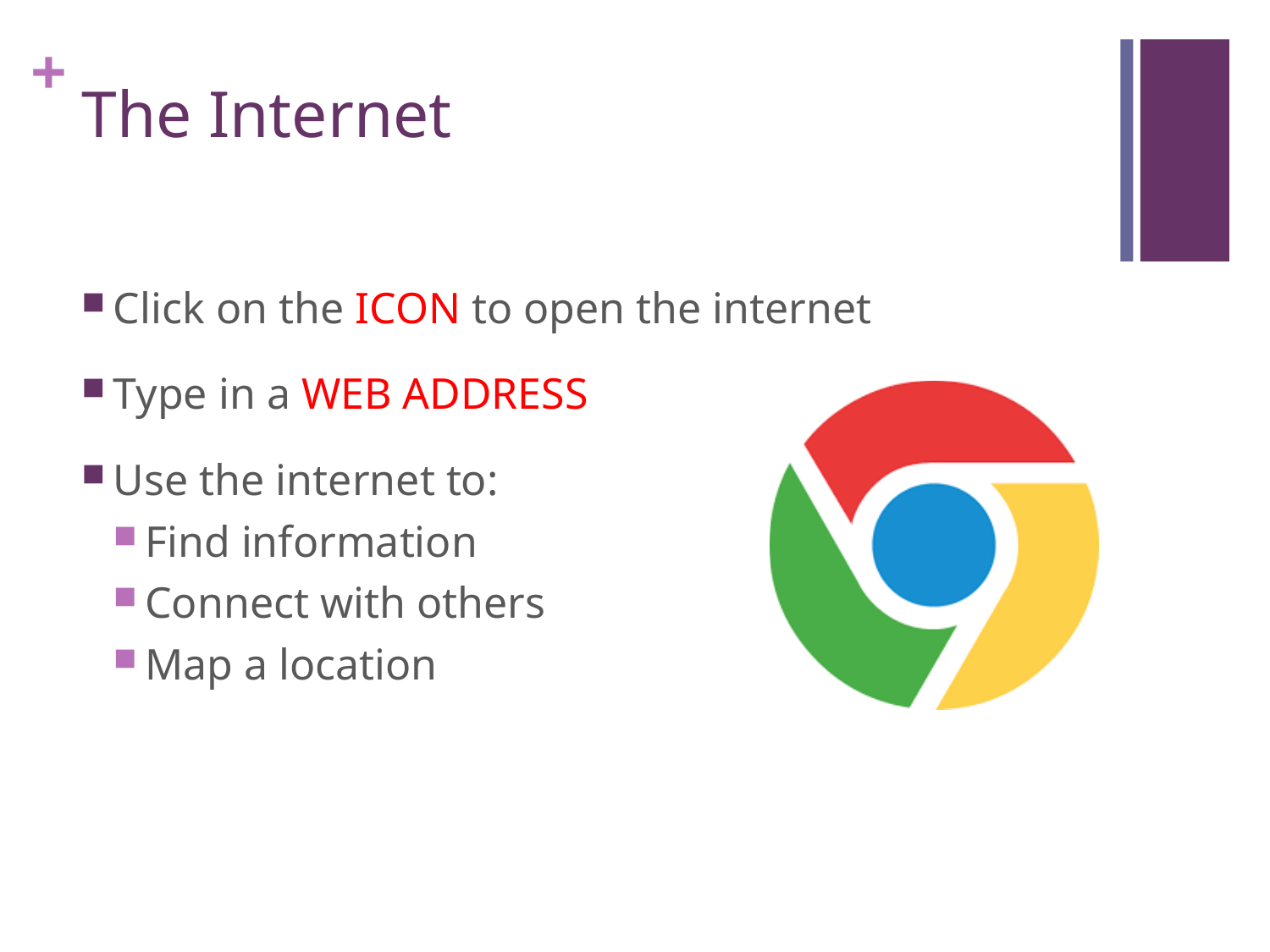

# The Internet
Click on the ICON to open the internet
Type in a WEB ADDRESS
Use the internet to:
Find information
Connect with others
Map a location

## Slide 5
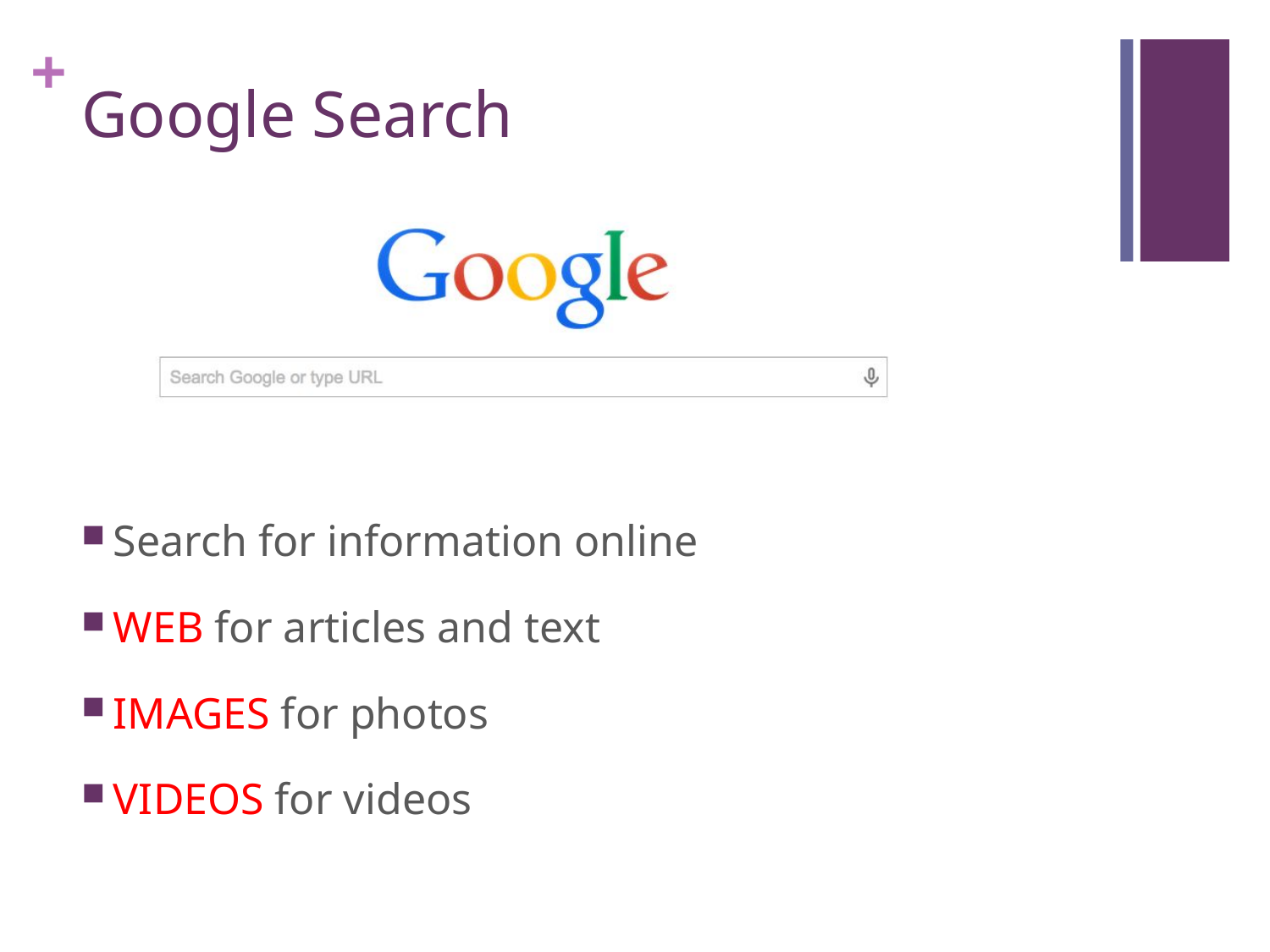

# Google Search
Search for information online
WEB for articles and text
IMAGES for photos
VIDEOS for videos

## Slide 6
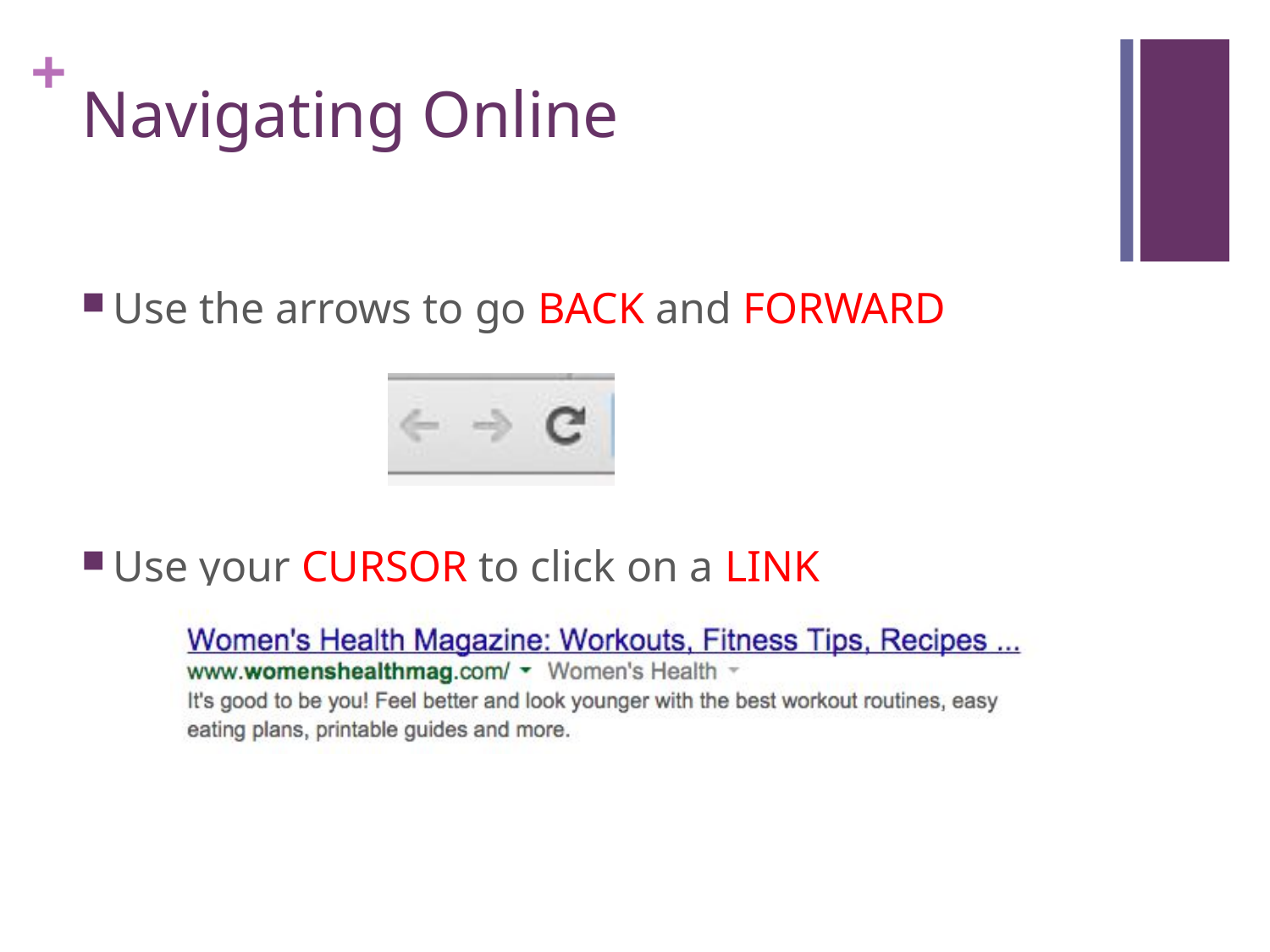

# Navigating Online
Use the arrows to go BACK and FORWARD
Use your CURSOR to click on a LINK

## Slide 7
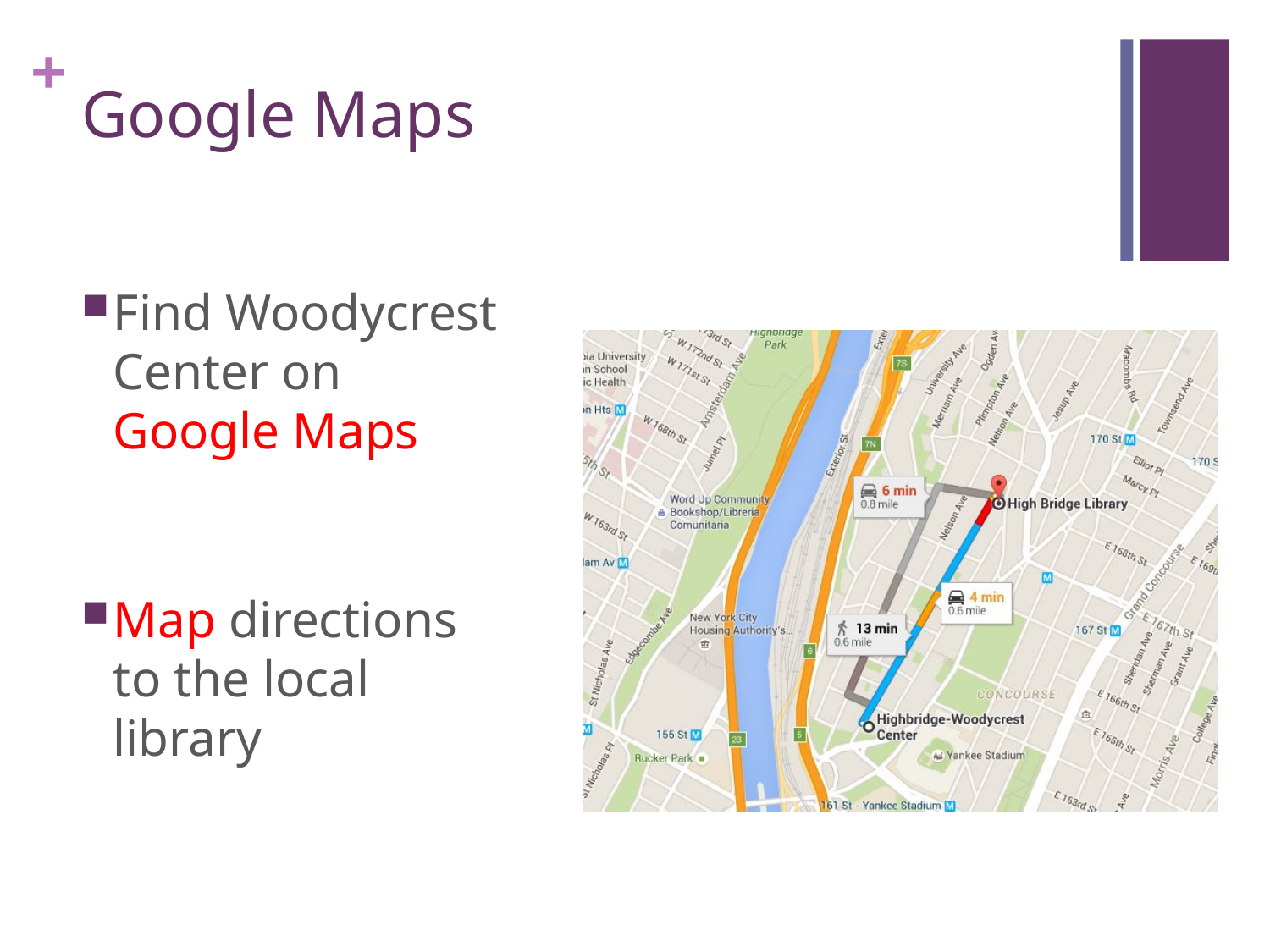

# Google Maps
Find Woodycrest Center on Google Maps
Map directions to the local library

## Slide 8
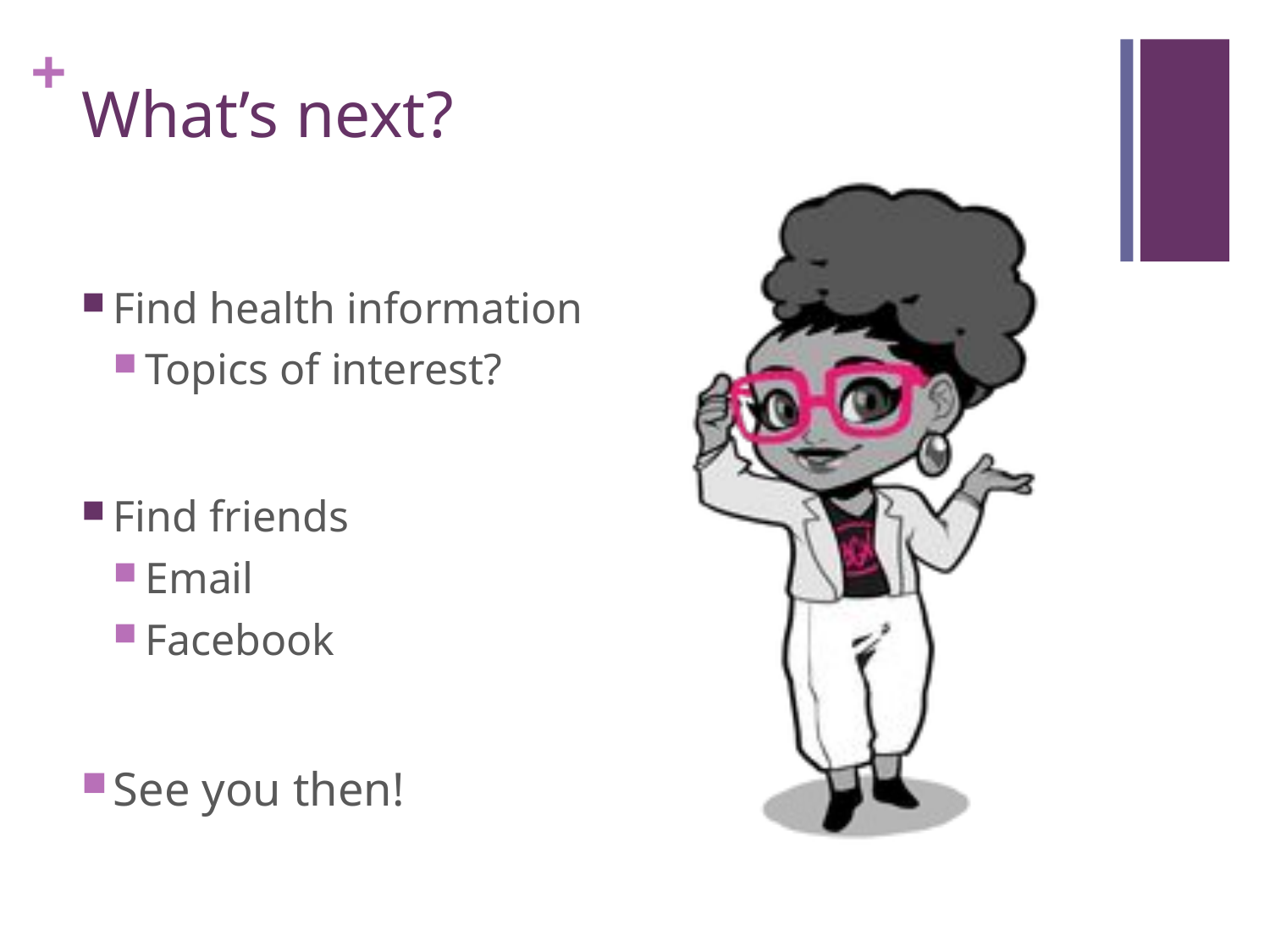

# What’s next?
Find health information
Topics of interest?
Find friends
Email
Facebook
See you then!
