# Supplementary material for: Get+Connected: Development and Pilot Testing of an Intervention to Improve Computer and Internet Attitudes and Internet Use Among Women Living With HIV
Source: JMIR Res Protoc. 2017 Mar 31;6(3):e50. doi: 10.2196/resprot.6391 (PMC5392213; doi:10.2196/resprot.6391)
Supplement: Multimedia Appendix 2 [file resprot_v6i3e_app2.ppt]

## Slide 1
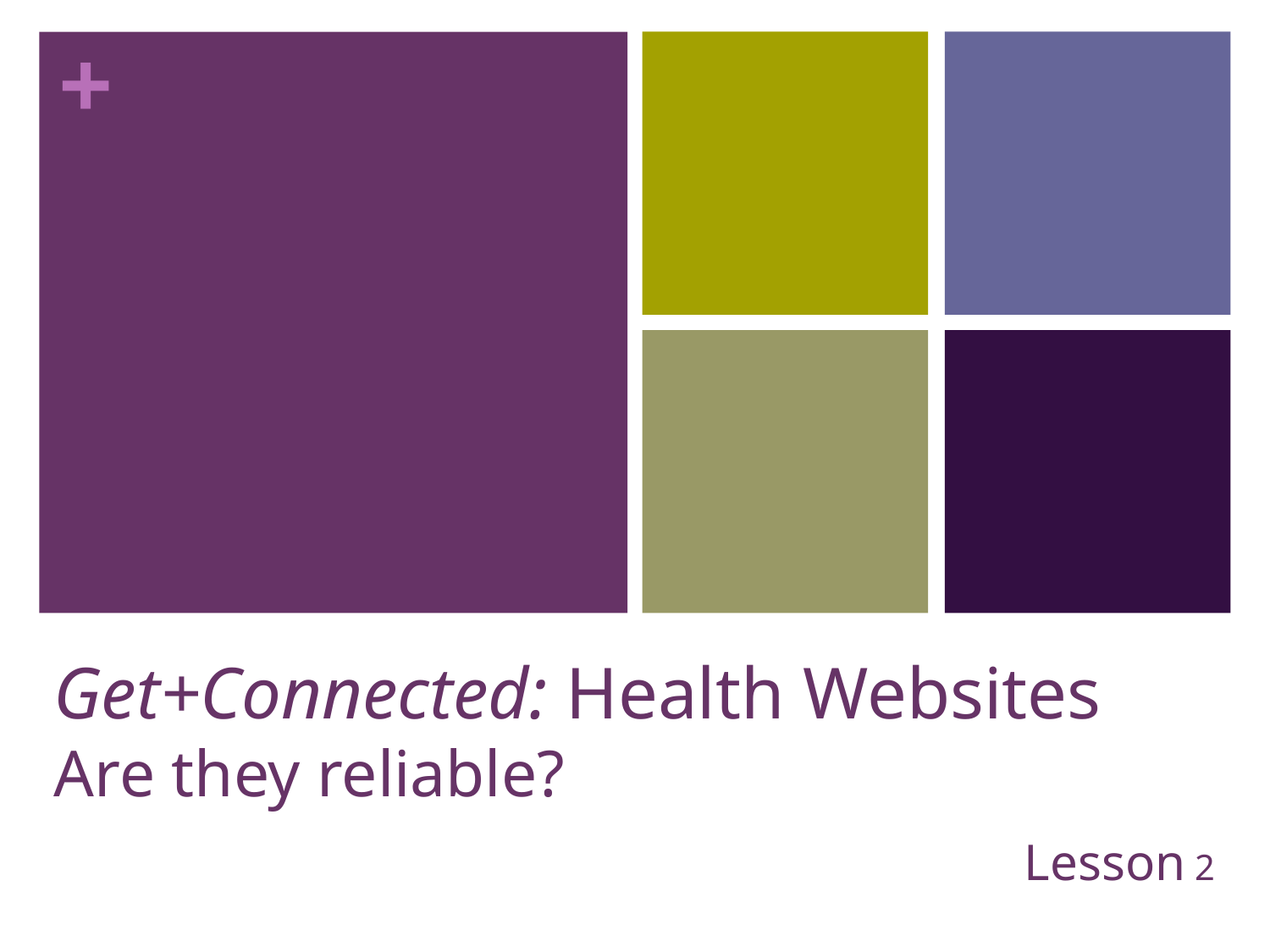

# Get+Connected: Health Websites Are they reliable?
Lesson 2

## Slide 2
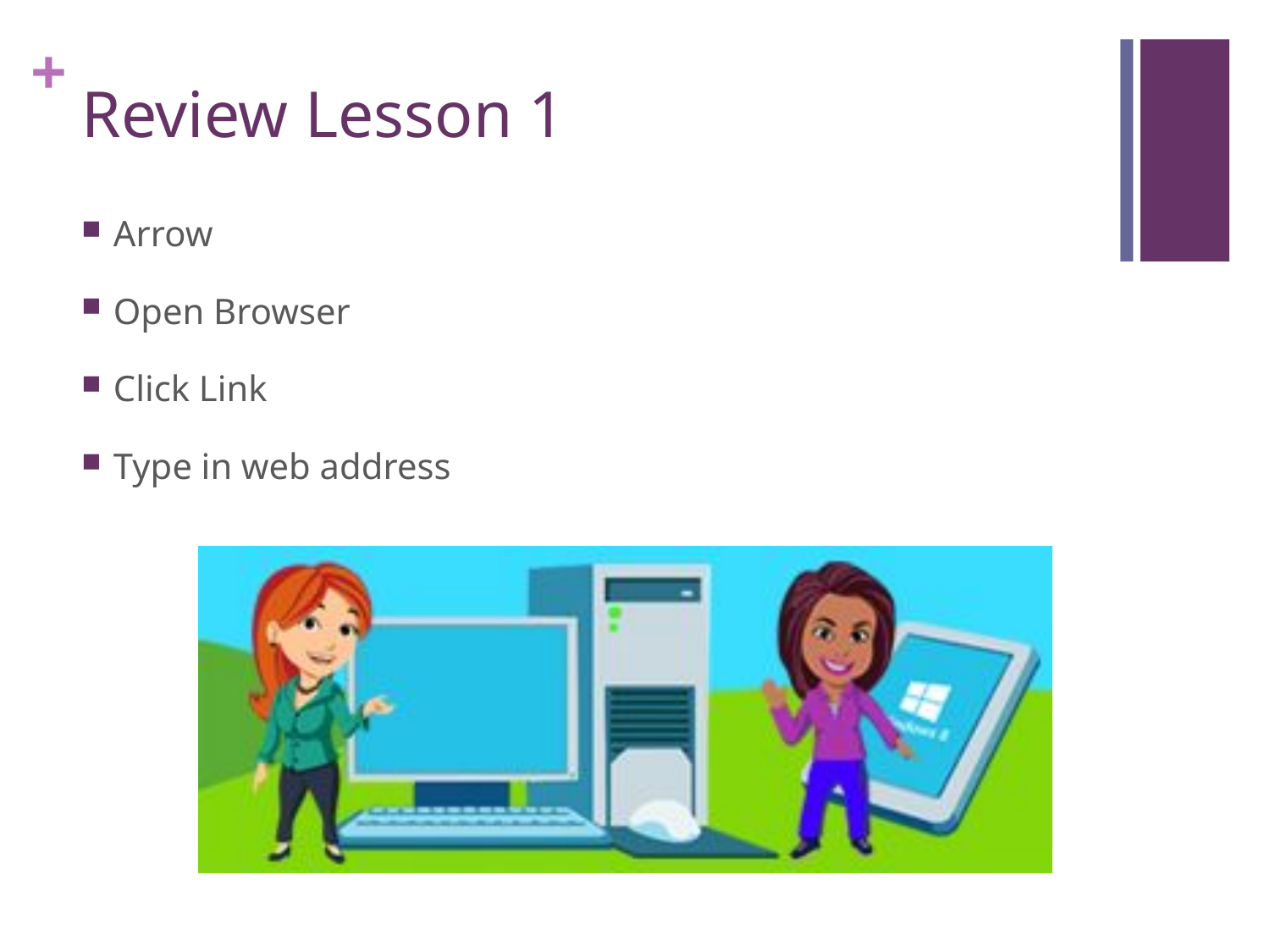

# Review Lesson 1
Arrow
Open Browser
Click Link
Type in web address

## Slide 3
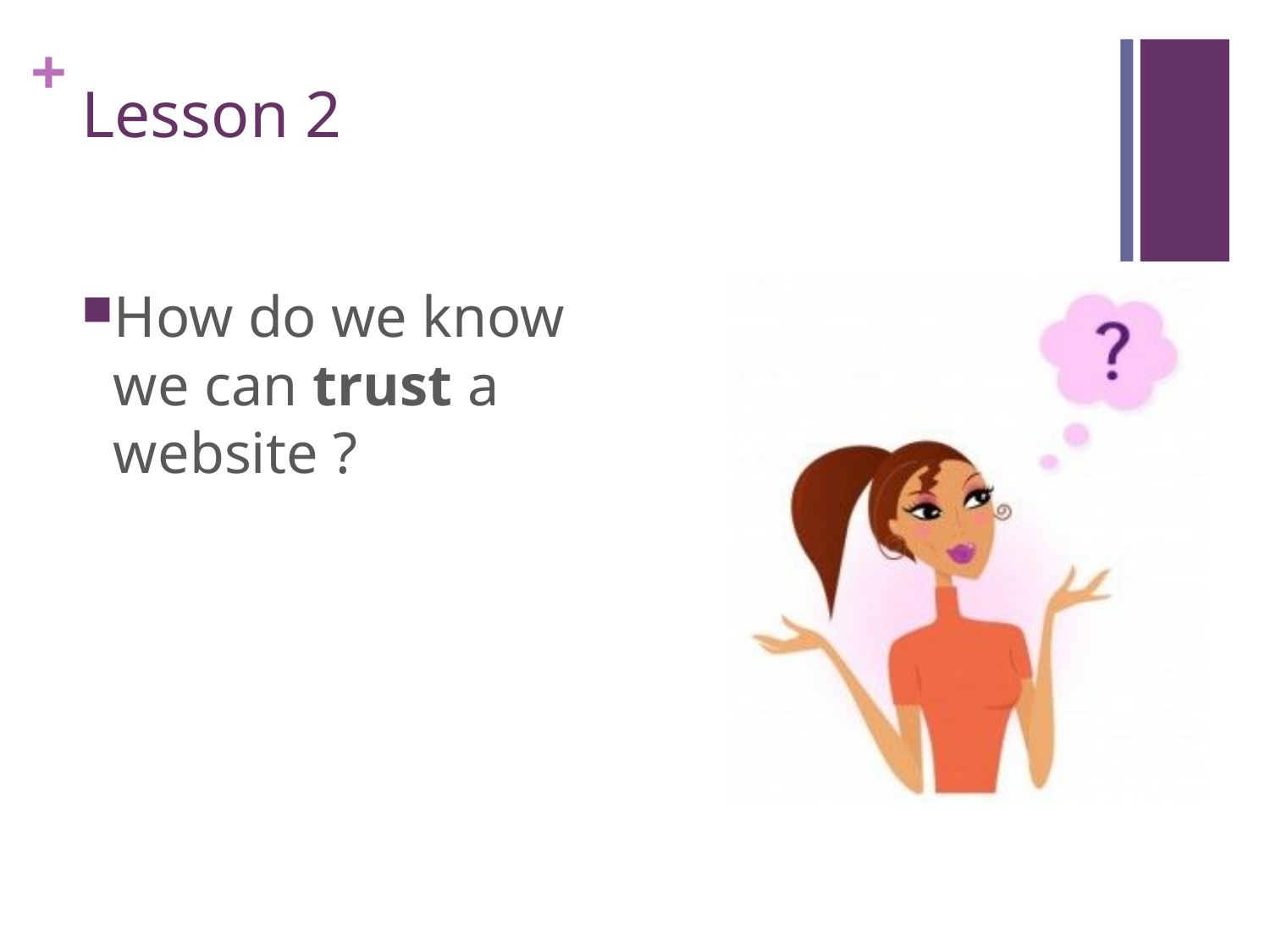

# Lesson 2
How do we know we can trust a website ?

## Slide 4
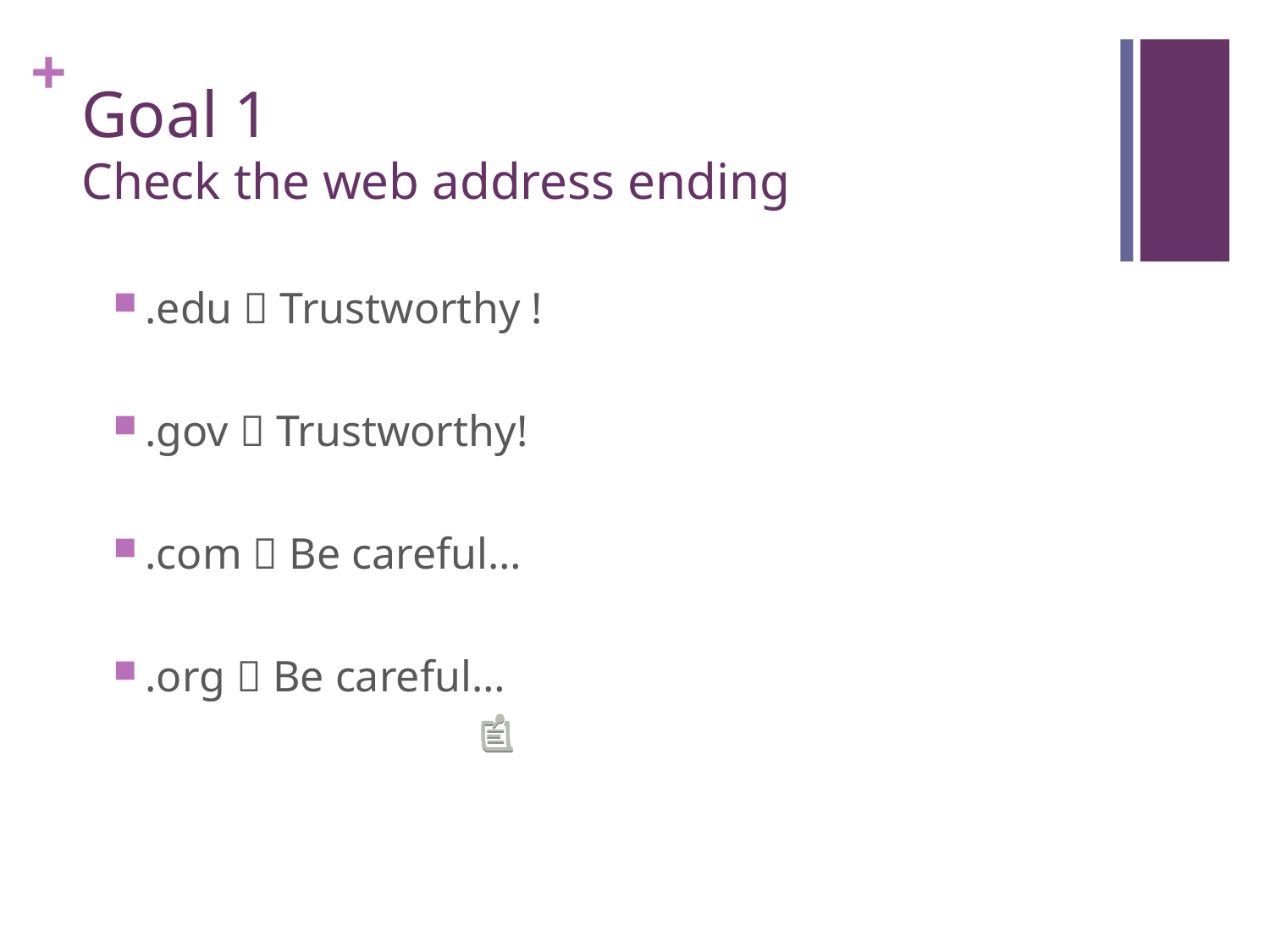

# Goal 1 Check the web address ending
.edu  Trustworthy !
.gov  Trustworthy!
.com  Be careful…
.org  Be careful…

## Slide 5
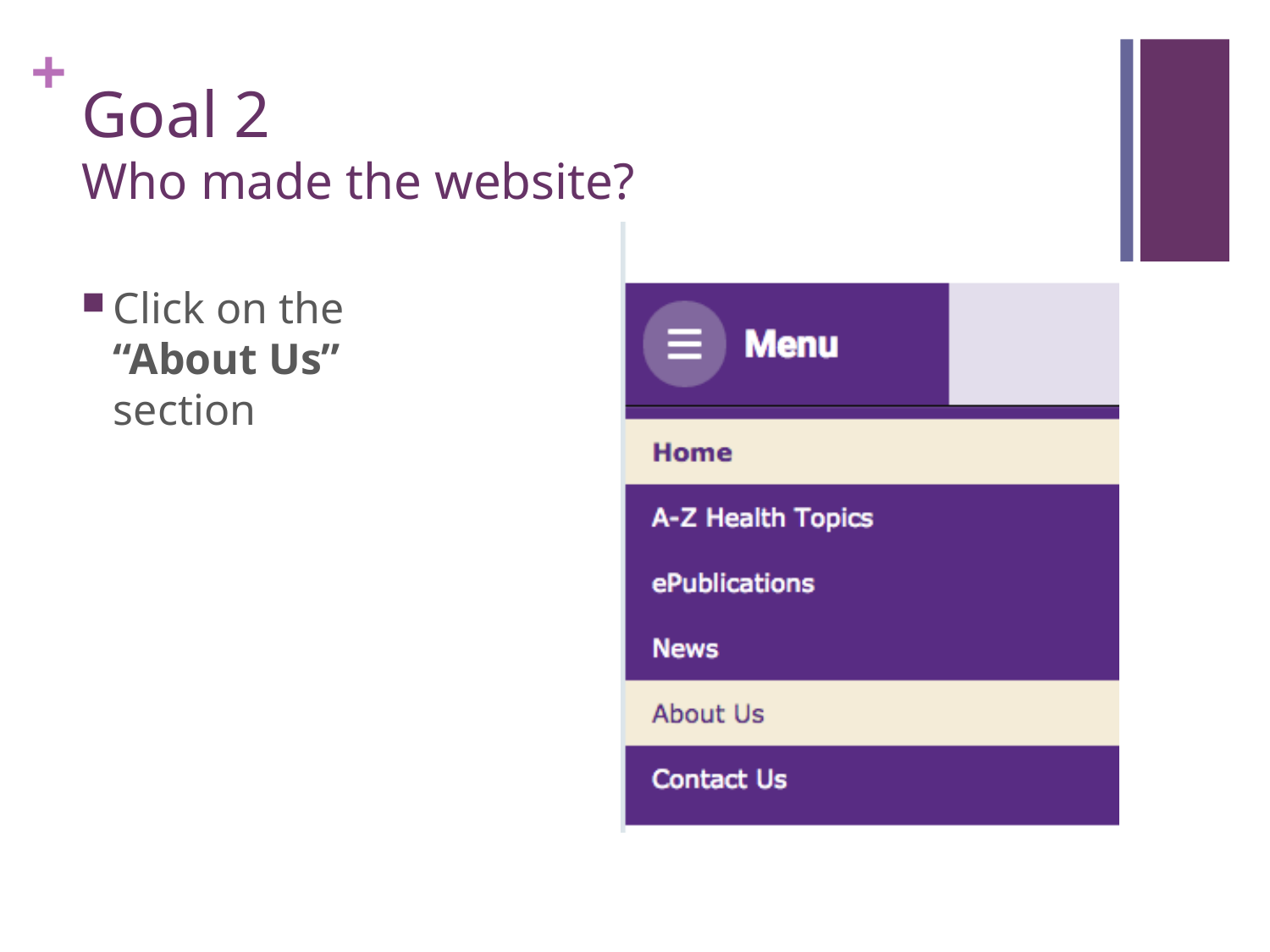

# Goal 2 Who made the website?
Click on the “About Us” section

## Slide 6
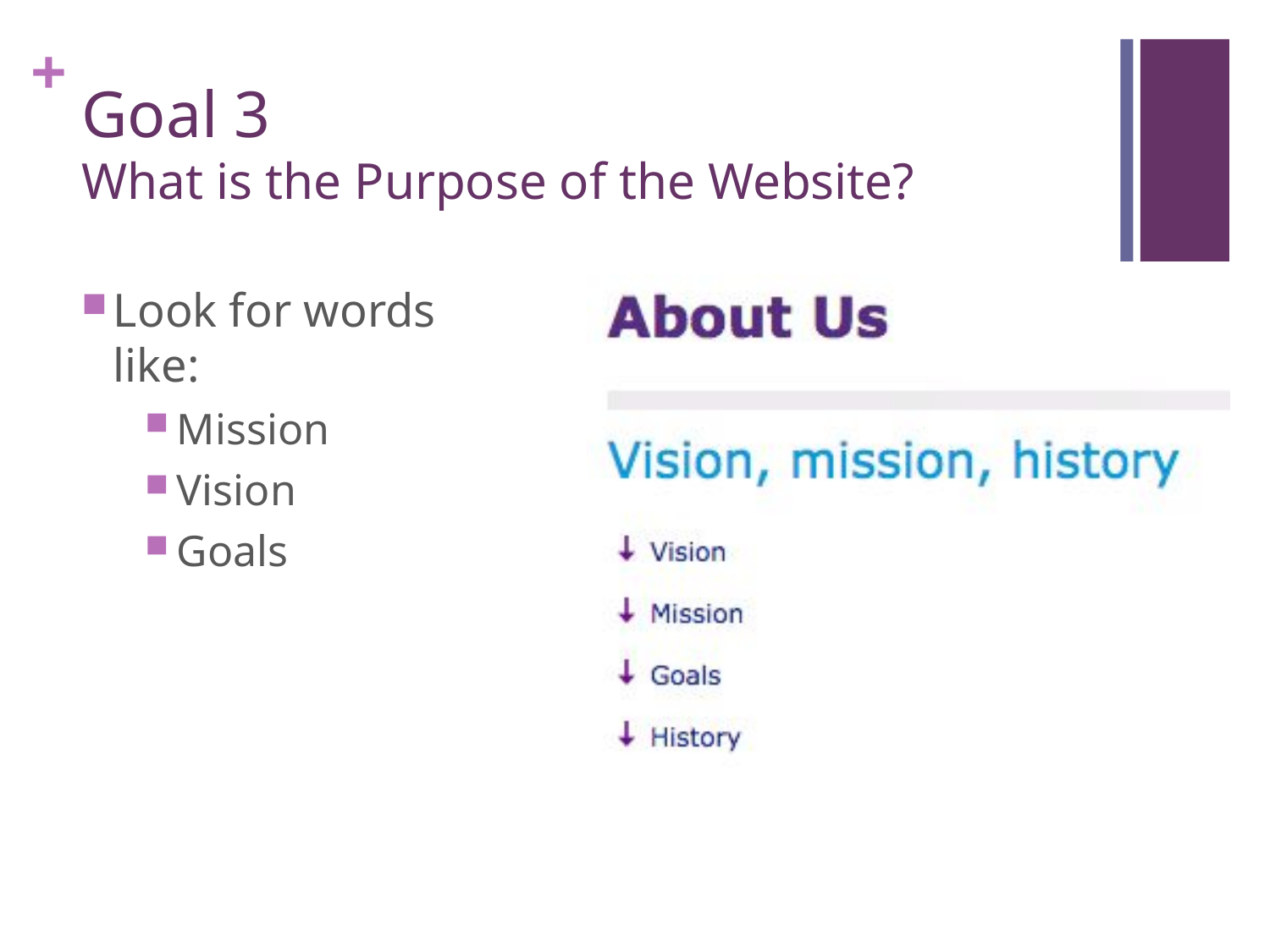

# Goal 3 What is the Purpose of the Website?
Look for words like:
Mission
Vision
Goals

## Slide 7
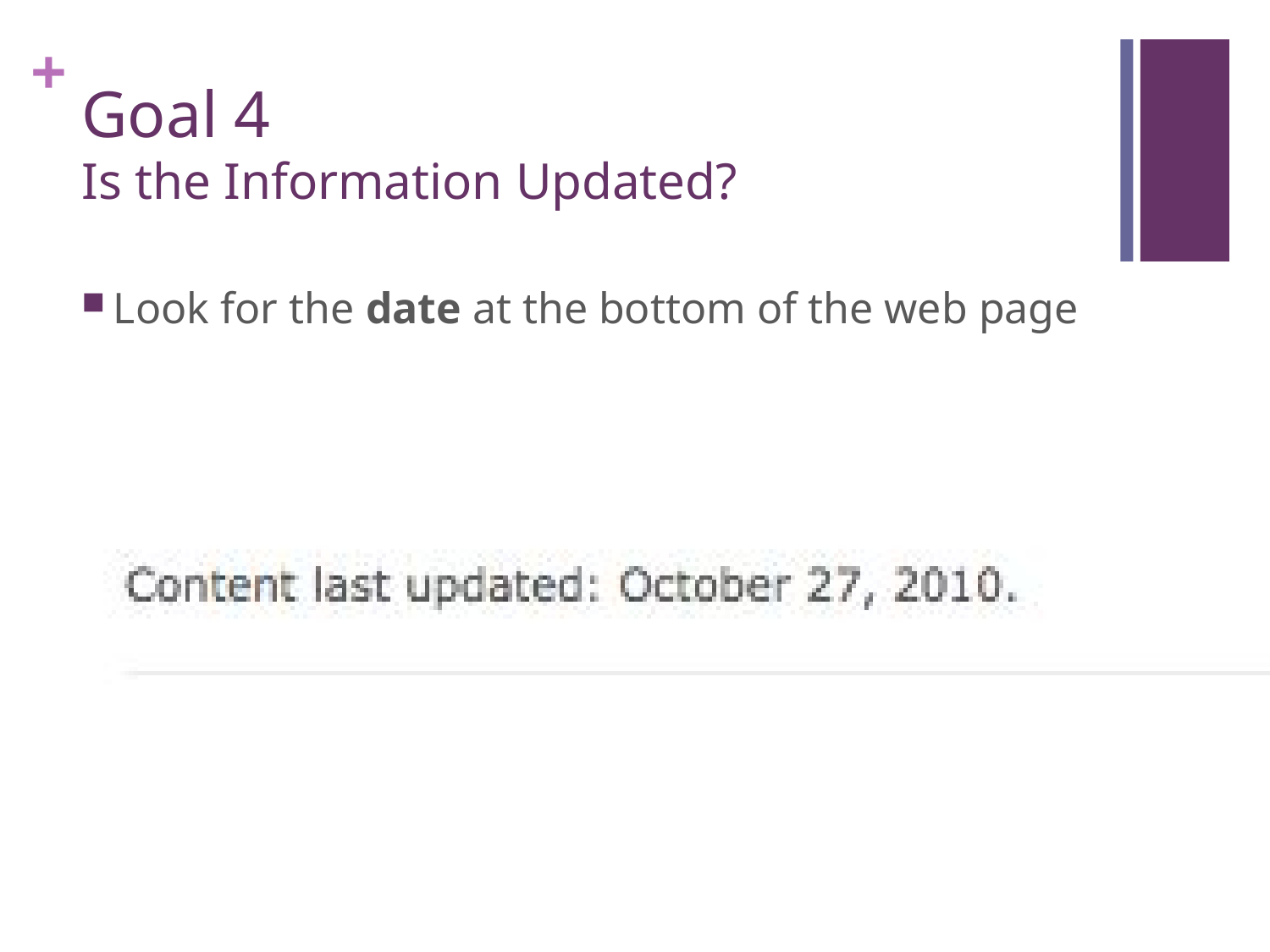

# Goal 4 Is the Information Updated?
Look for the date at the bottom of the web page

## Slide 8
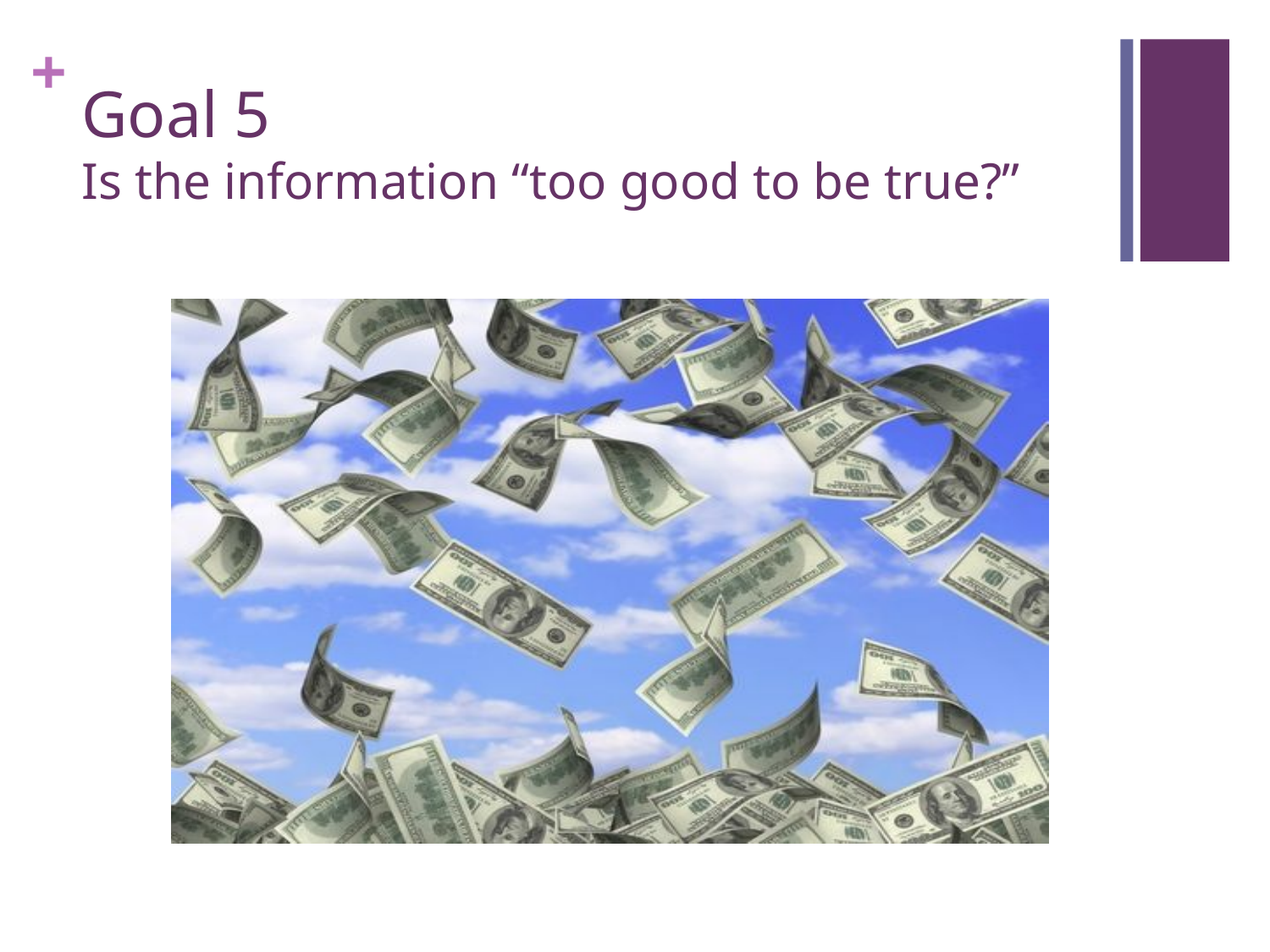

# Goal 5 Is the information “too good to be true?”

## Slide 9
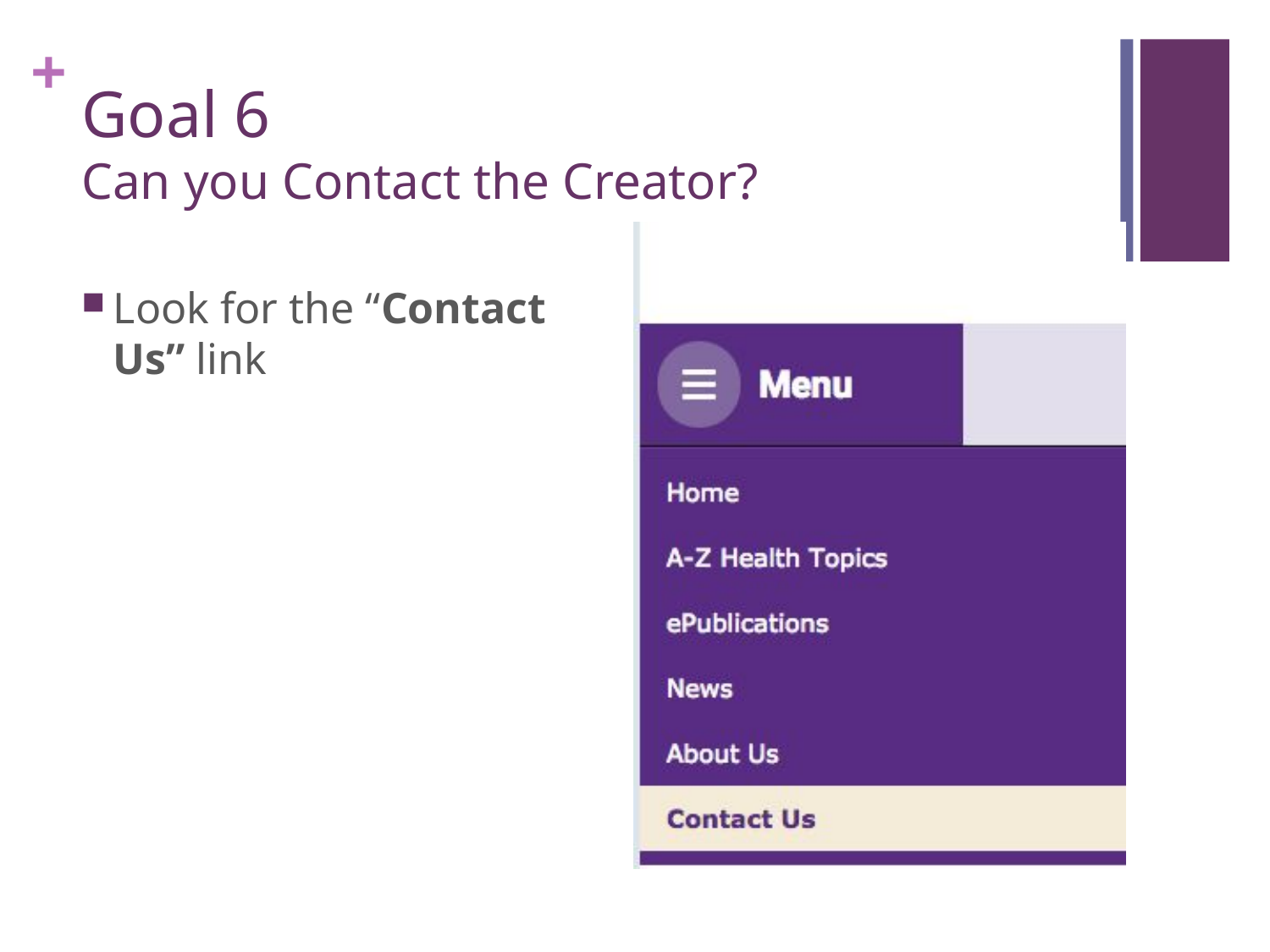

# Goal 6 Can you Contact the Creator?
Look for the “Contact Us” link

## Slide 10
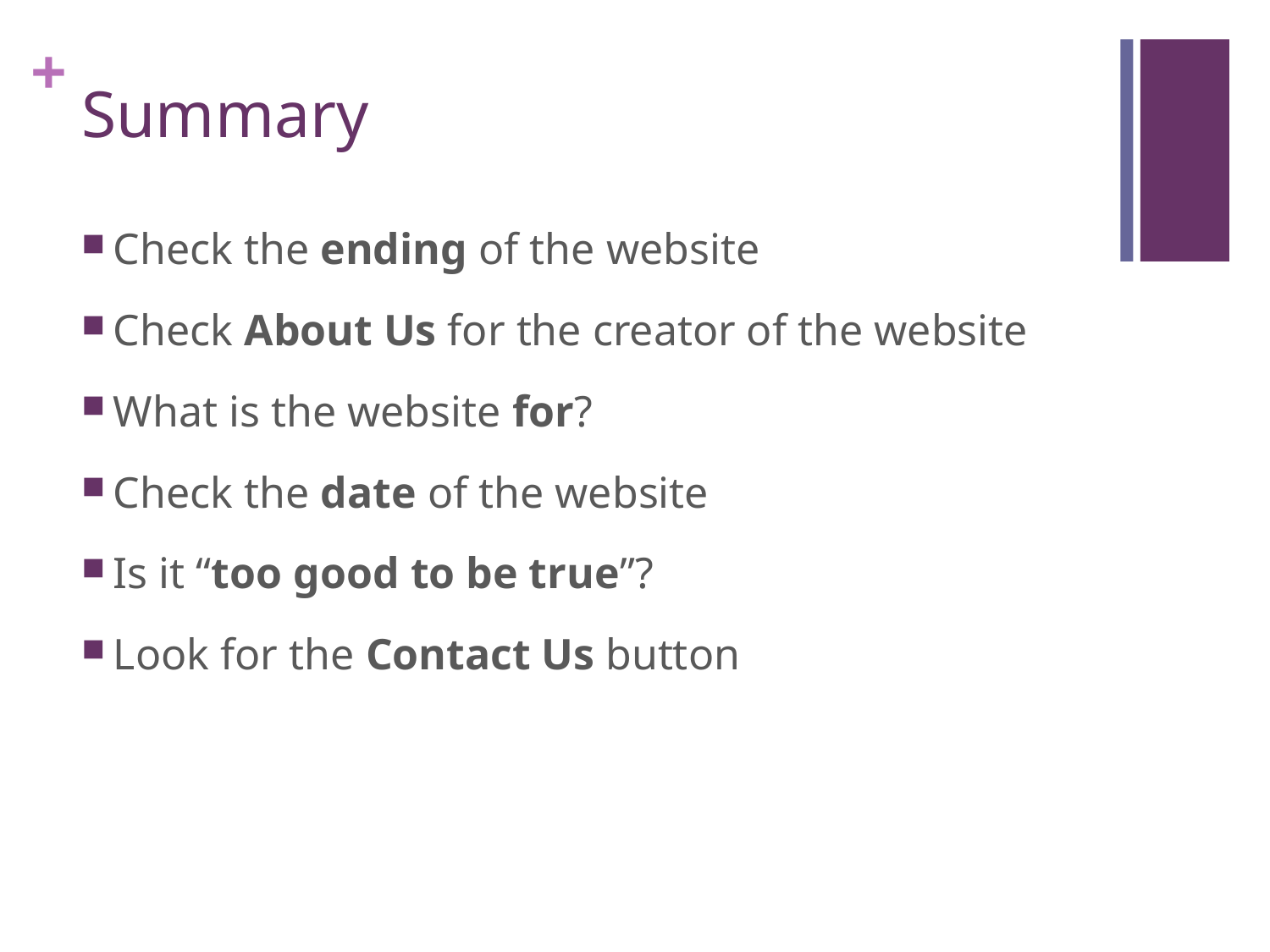

# Summary
Check the ending of the website
Check About Us for the creator of the website
What is the website for?
Check the date of the website
Is it “too good to be true”?
Look for the Contact Us button
