# Supplementary material for: Get+Connected: Development and Pilot Testing of an Intervention to Improve Computer and Internet Attitudes and Internet Use Among Women Living With HIV
Source: JMIR Res Protoc. 2017 Mar 31;6(3):e50. doi: 10.2196/resprot.6391 (PMC5392213; doi:10.2196/resprot.6391)
Supplement: Multimedia Appendix 3 [file resprot_v6i3e_app3.ppt]

## Slide 1
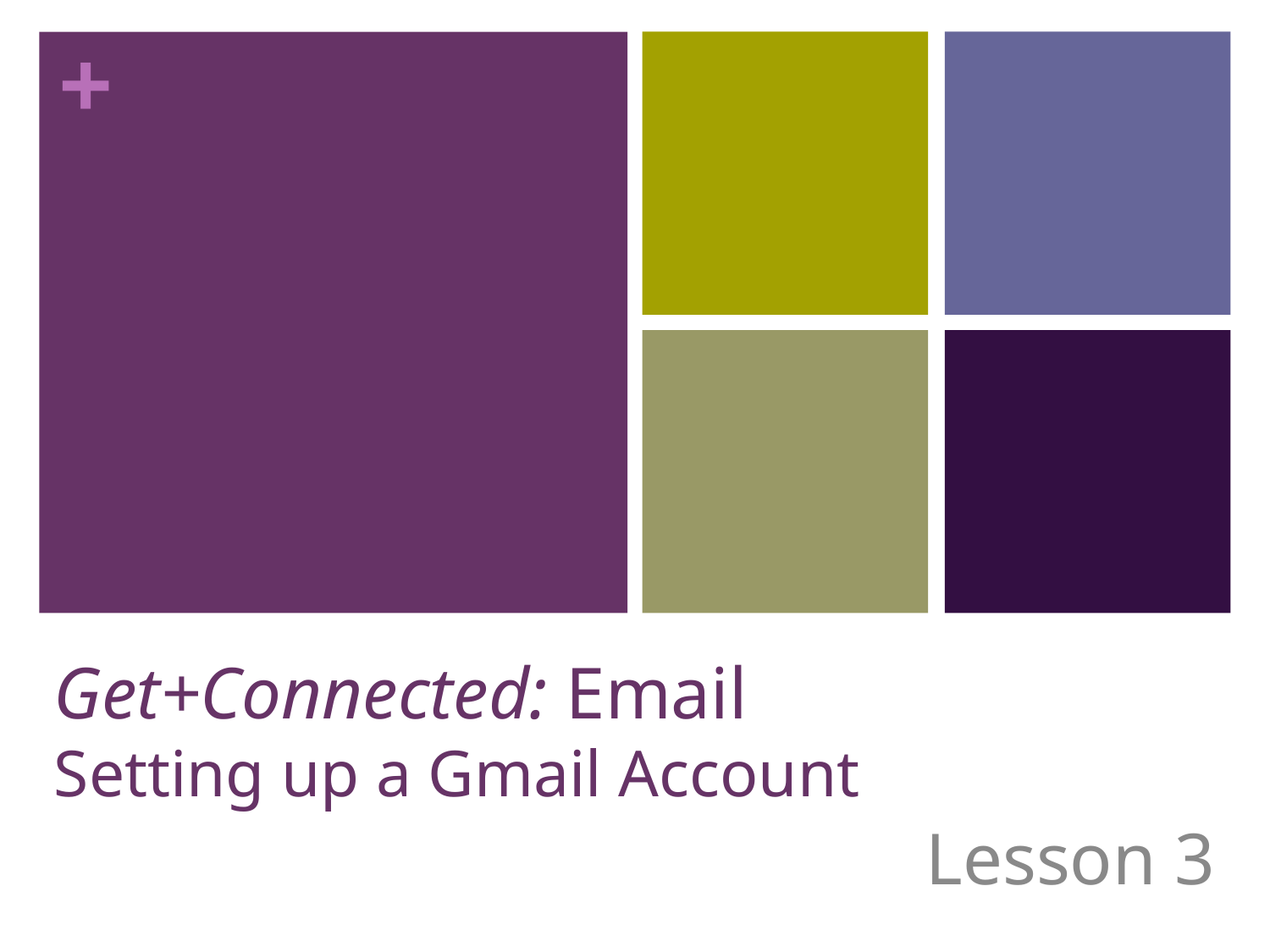

# Get+Connected: Email Setting up a Gmail Account
Lesson 3

## Slide 2
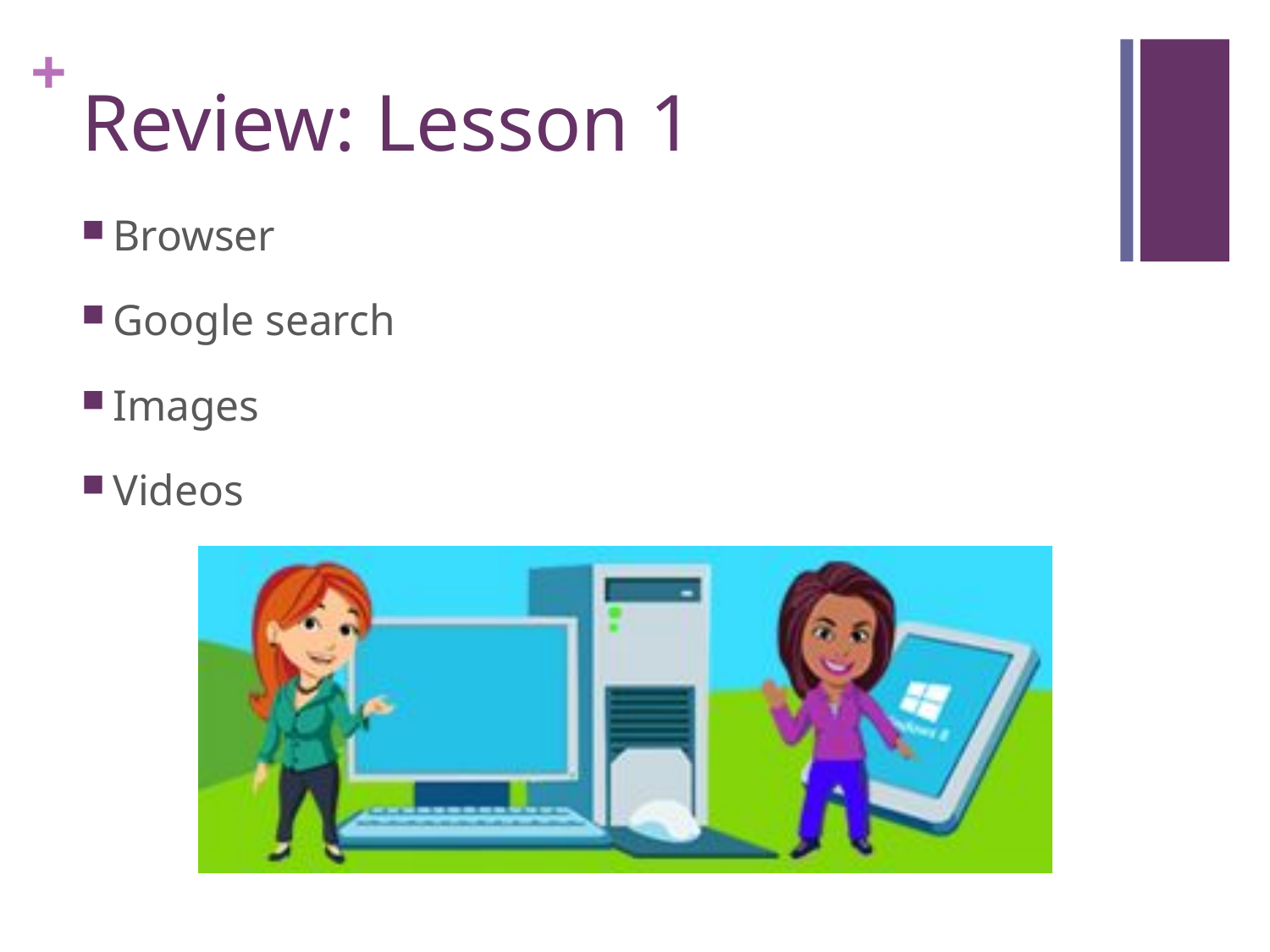

# Review: Lesson 1
Browser
Google search
Images
Videos

## Slide 3
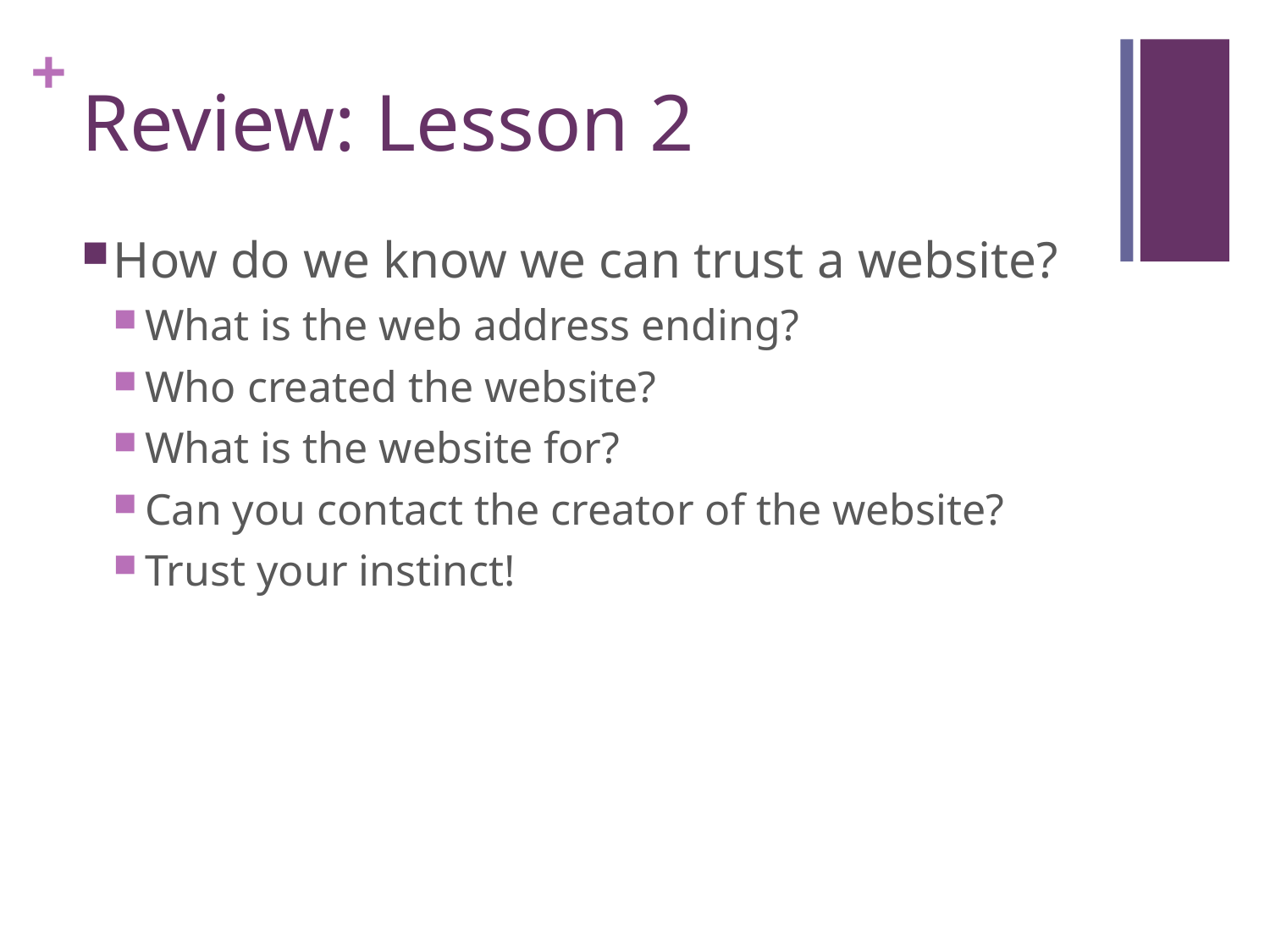

# Review: Lesson 2
How do we know we can trust a website?
What is the web address ending?
Who created the website?
What is the website for?
Can you contact the creator of the website?
Trust your instinct!

## Slide 4
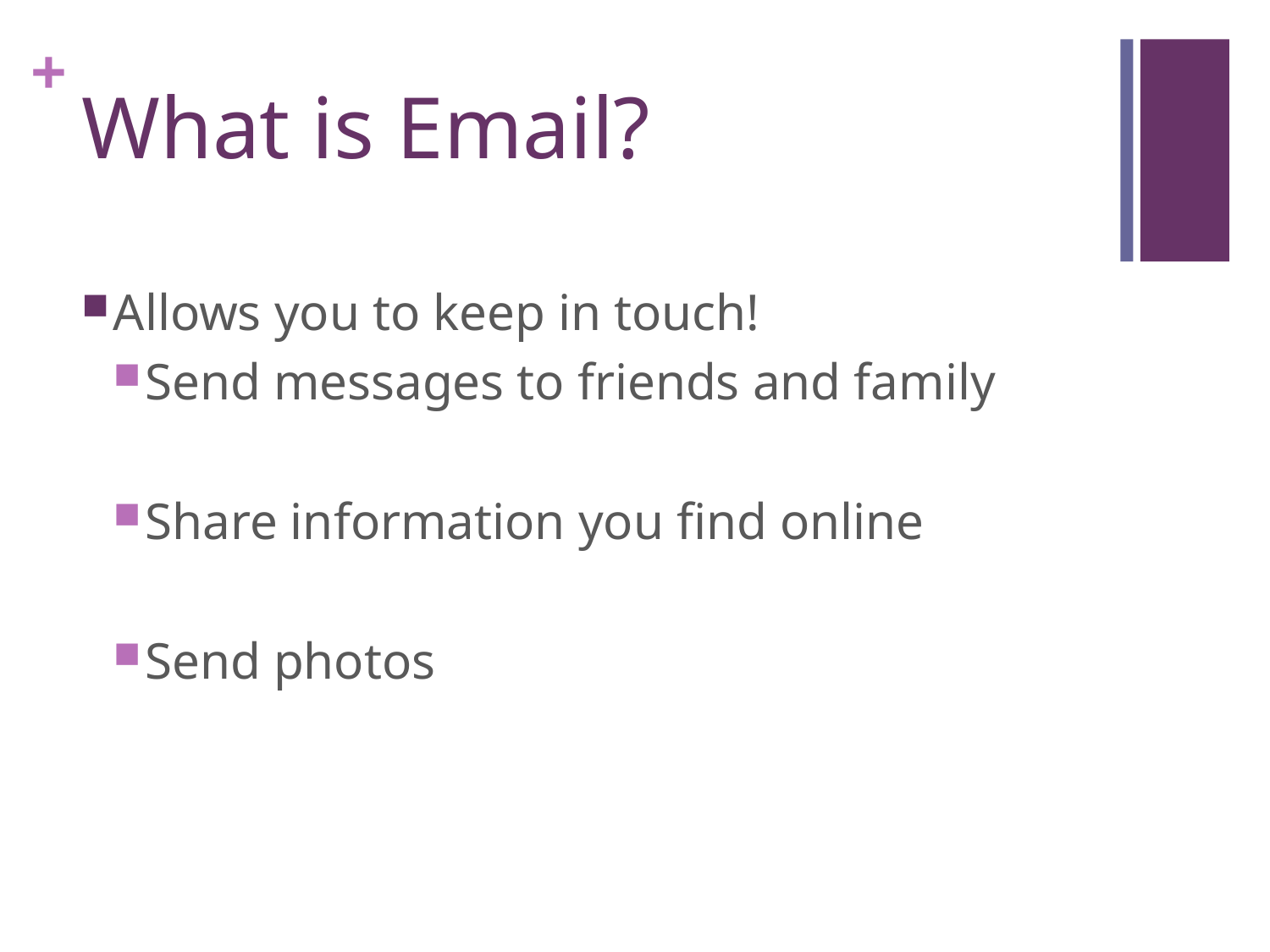

# What is Email?
Allows you to keep in touch!
Send messages to friends and family
Share information you find online
Send photos

## Slide 5
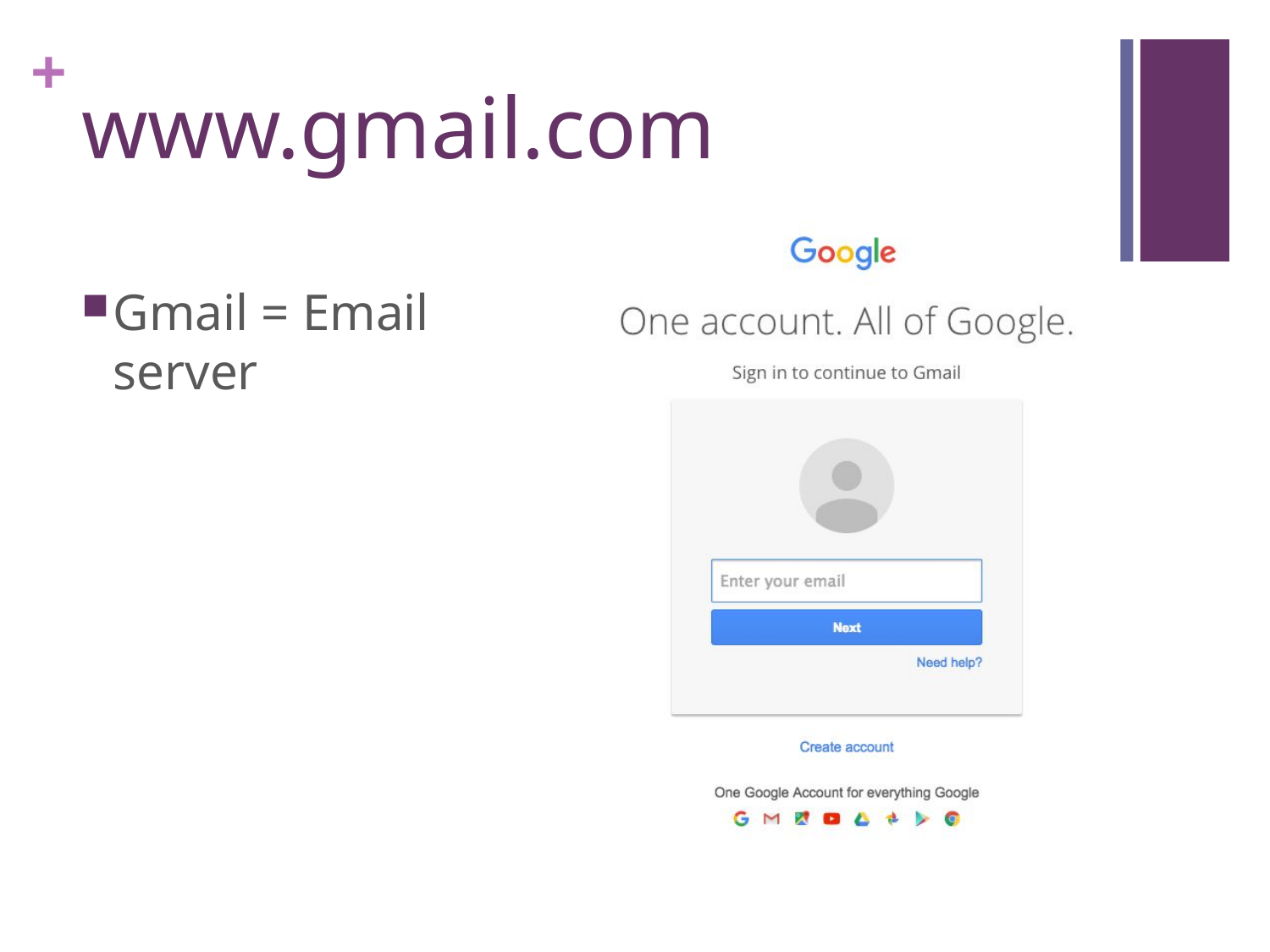

# www.gmail.com
Gmail = Email server

## Slide 6
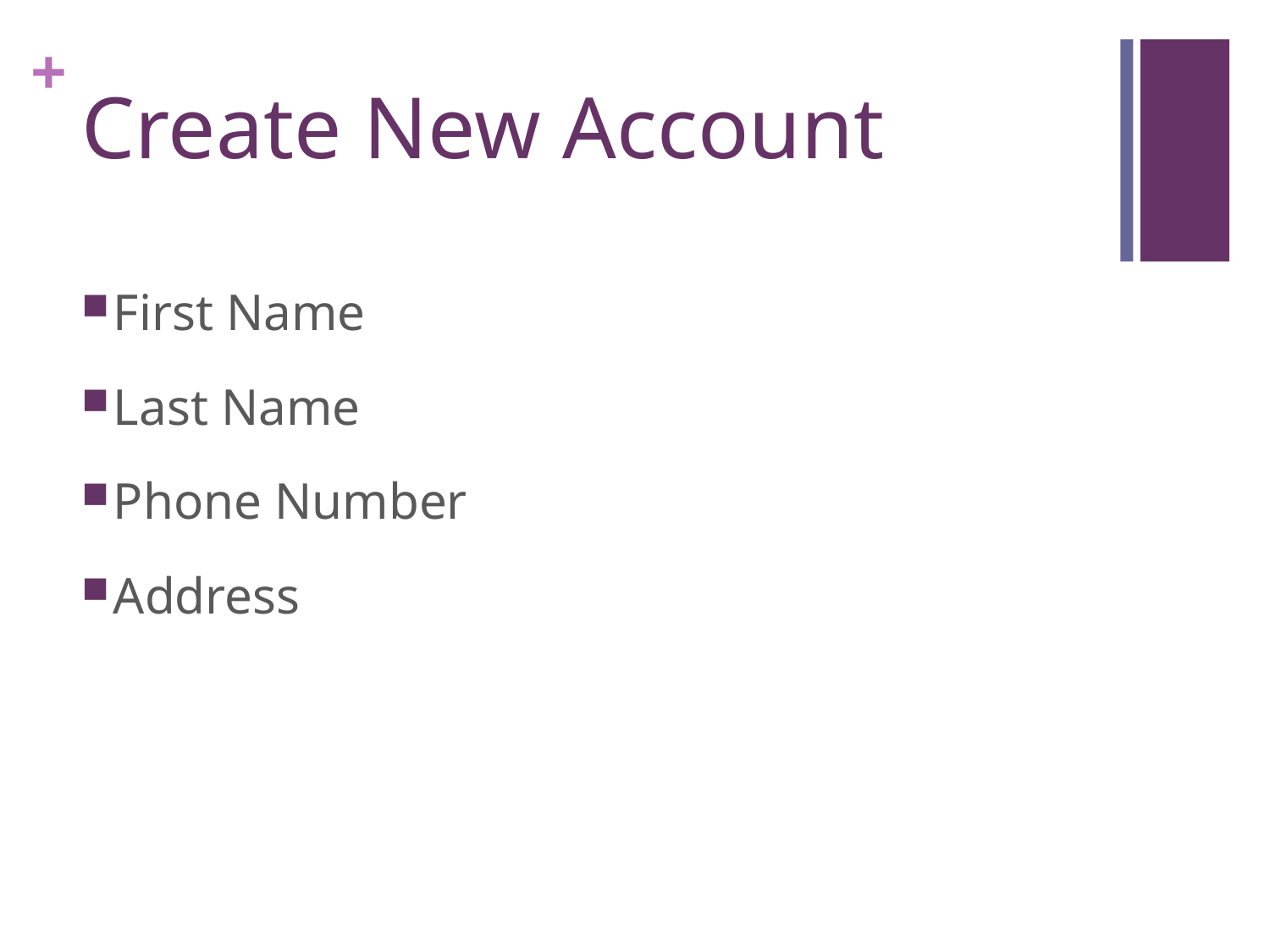

# Create New Account
First Name
Last Name
Phone Number
Address

## Slide 7
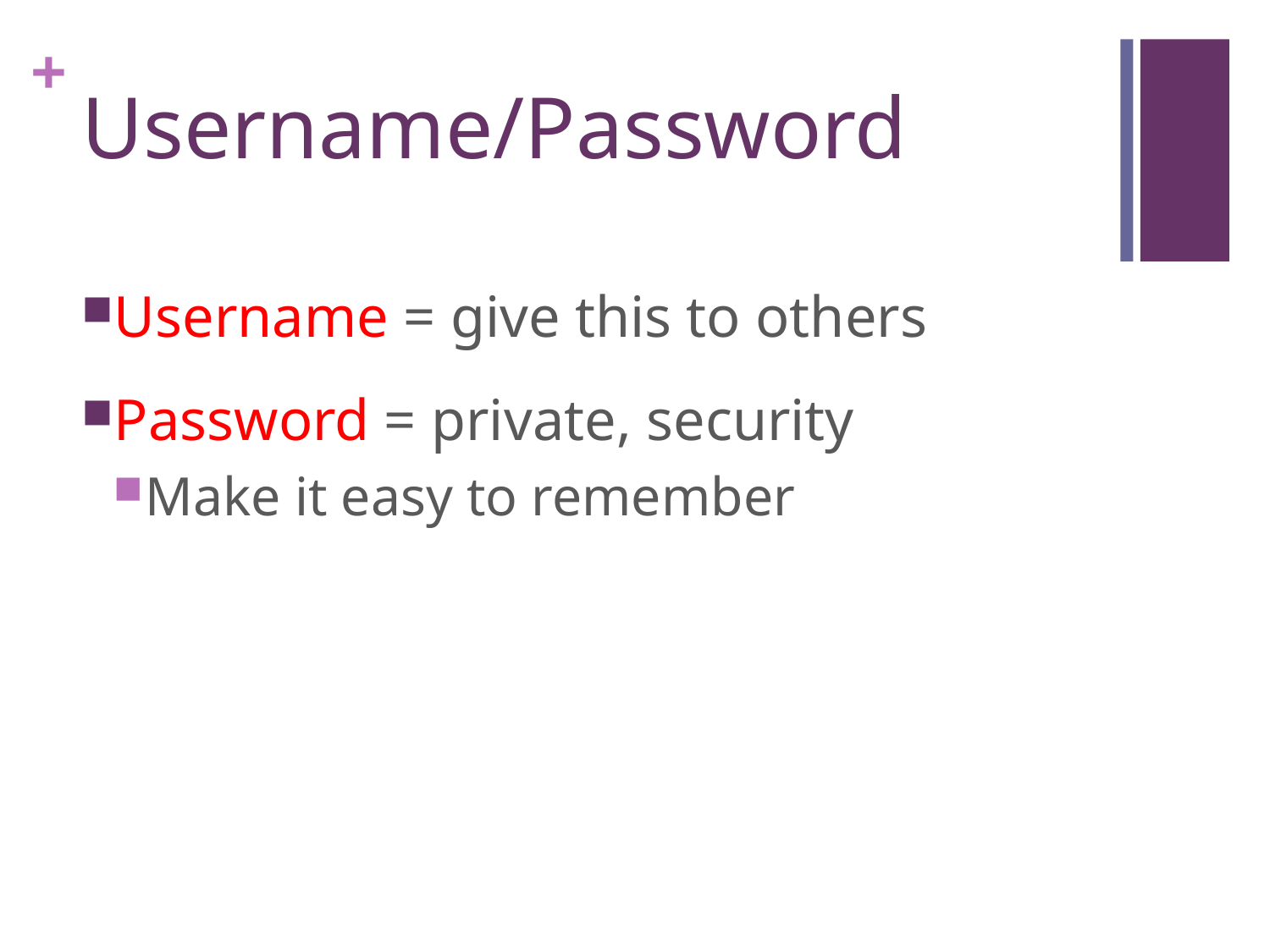

# Username/Password
Username = give this to others
Password = private, security
Make it easy to remember

## Slide 8
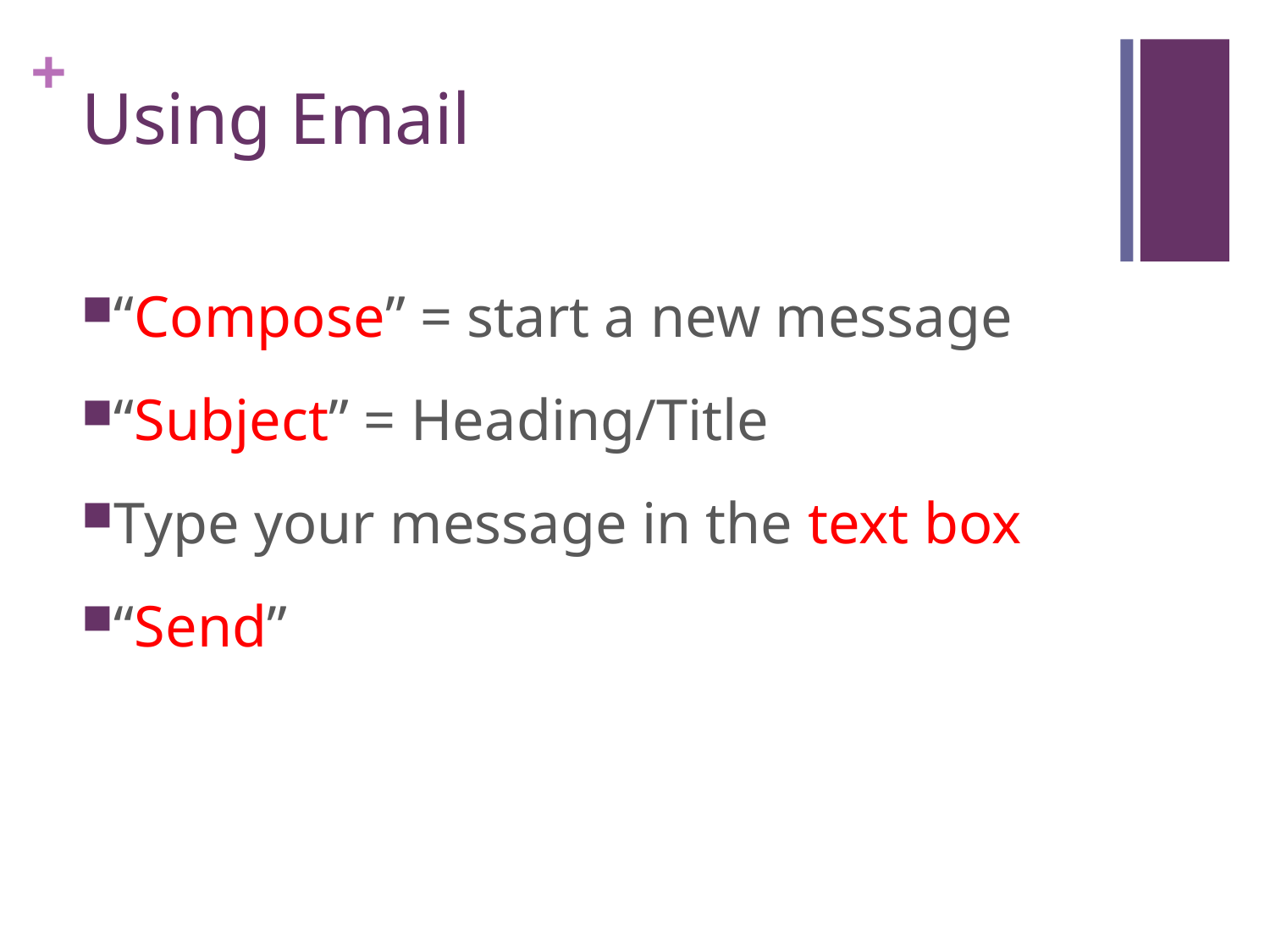

# Using Email
“Compose” = start a new message
“Subject” = Heading/Title
Type your message in the text box
“Send”

## Slide 9
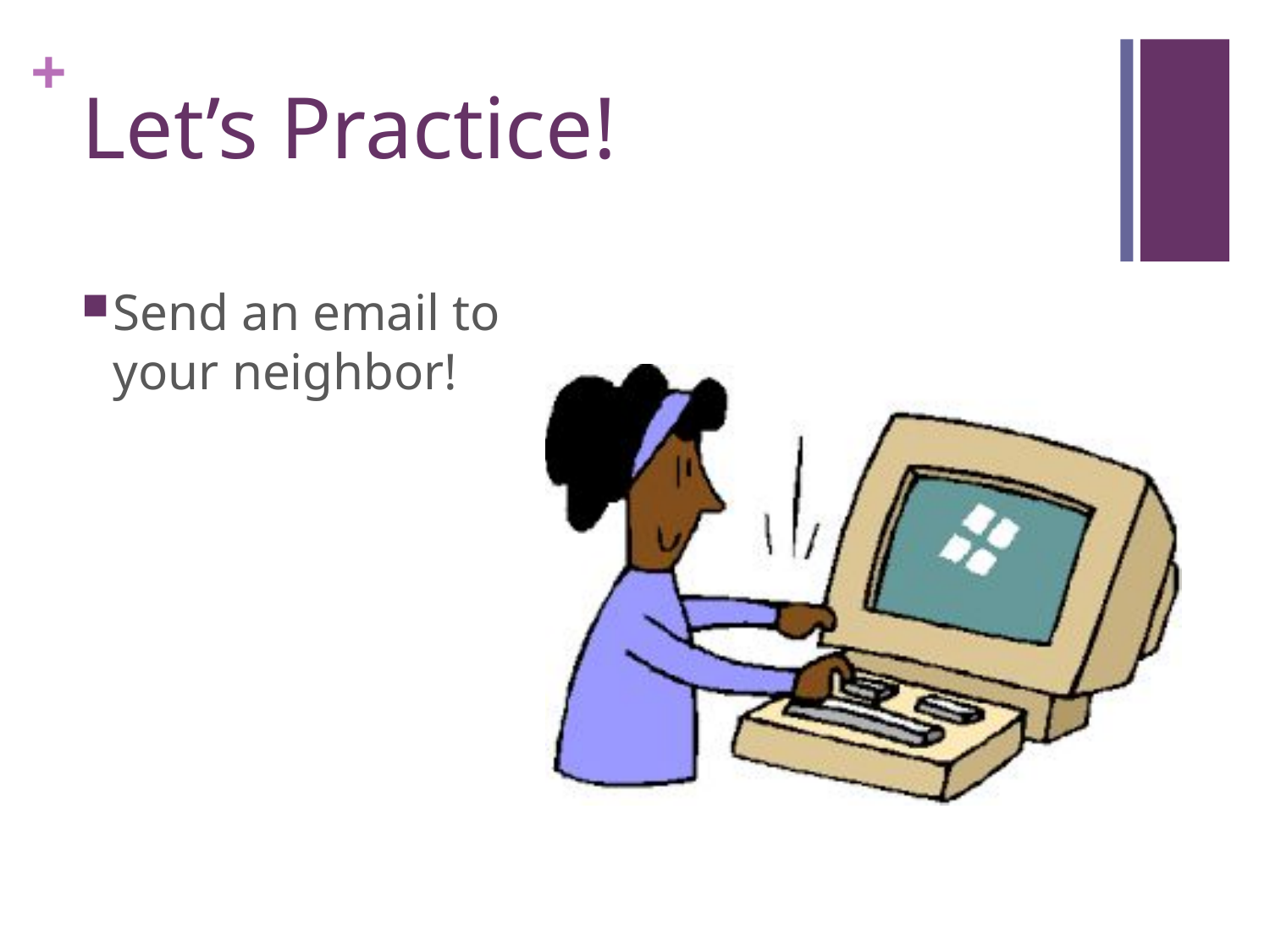

# Let’s Practice!
Send an email to your neighbor!

## Slide 10
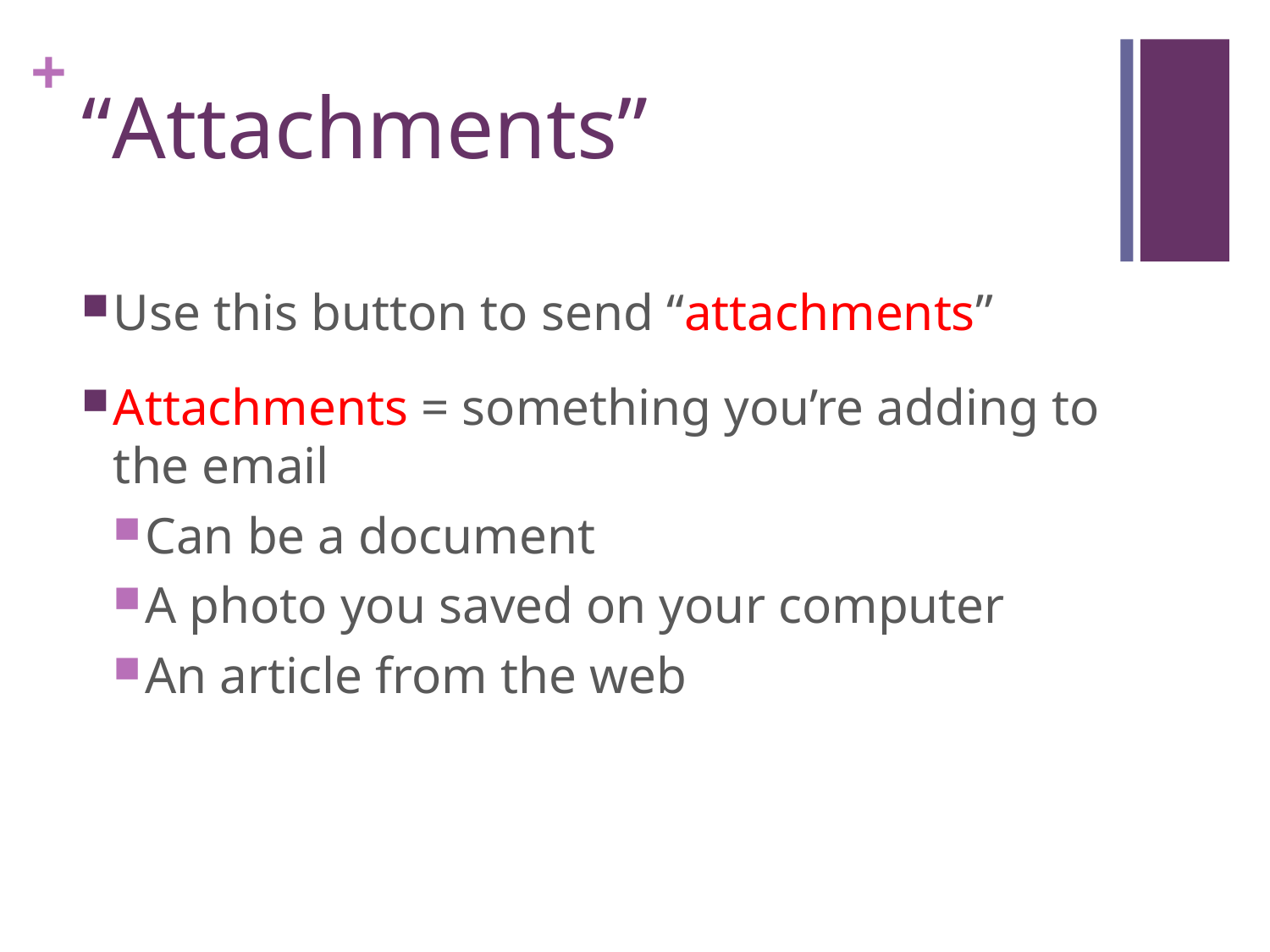

# “Attachments”
Use this button to send “attachments”
Attachments = something you’re adding to the email
Can be a document
A photo you saved on your computer
An article from the web

## Slide 11
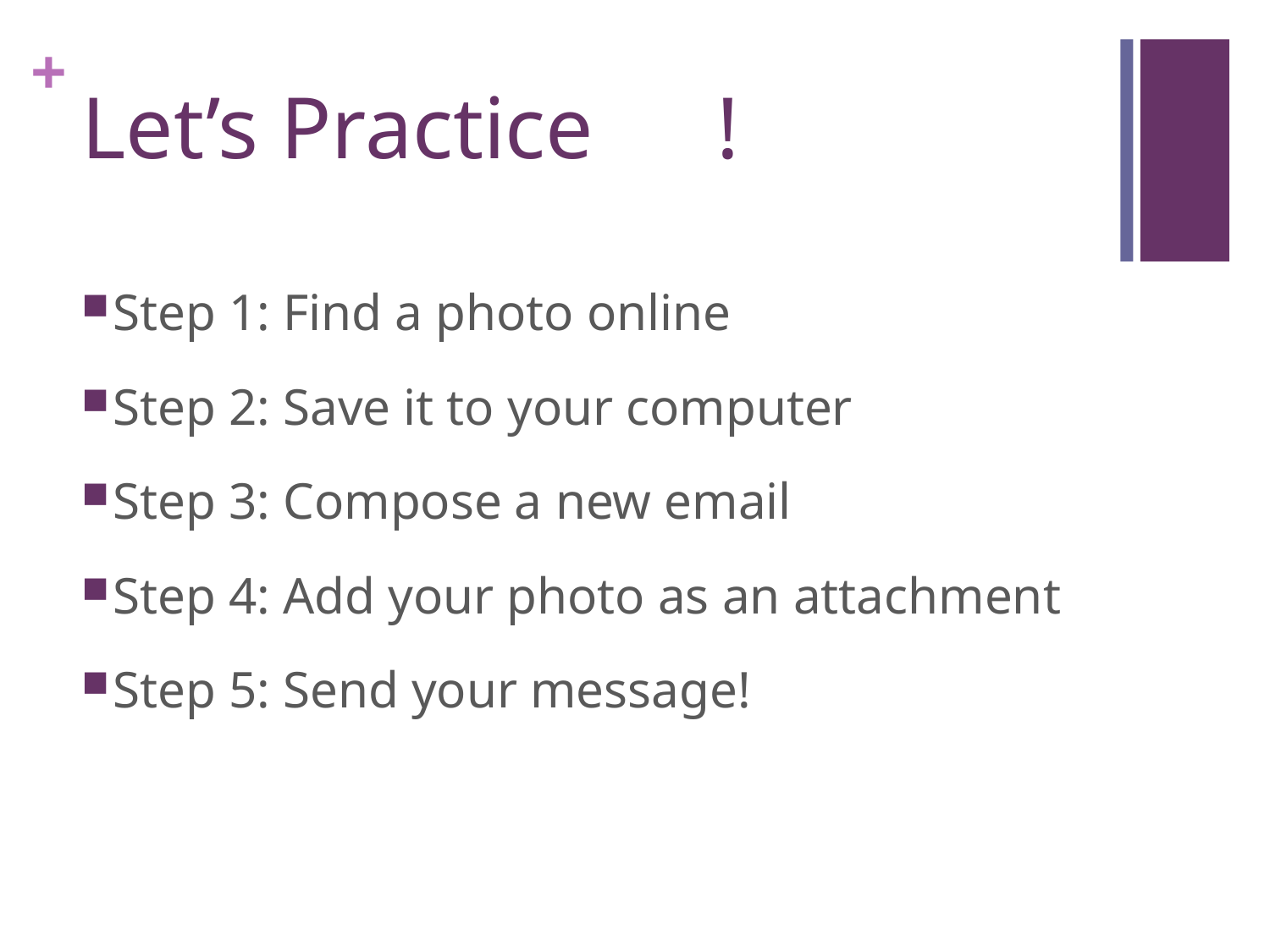

# Let’s Practice	!
Step 1: Find a photo online
Step 2: Save it to your computer
Step 3: Compose a new email
Step 4: Add your photo as an attachment
Step 5: Send your message!

## Slide 12
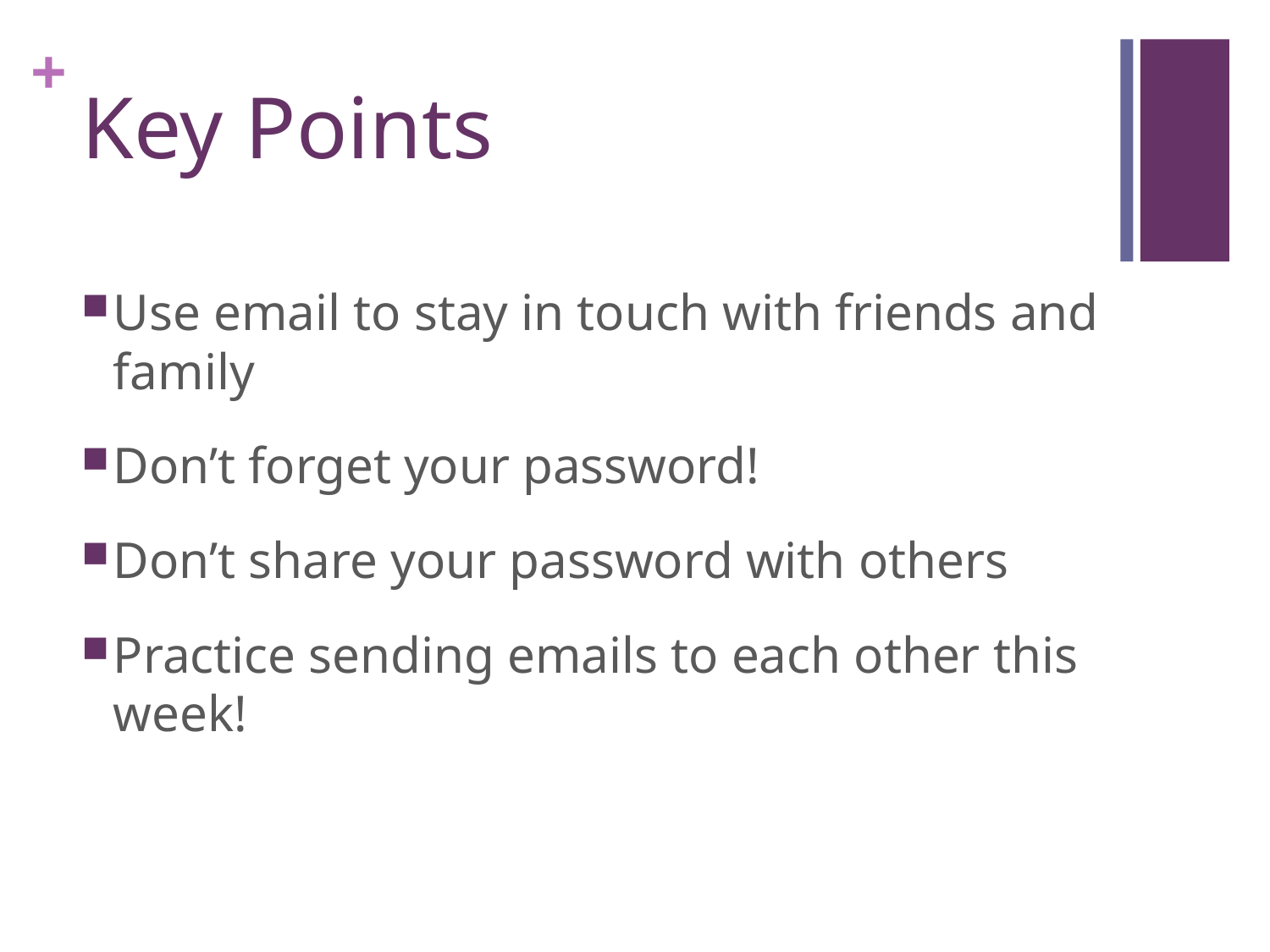

# Key Points
Use email to stay in touch with friends and family
Don’t forget your password!
Don’t share your password with others
Practice sending emails to each other this week!
