# Supplementary material for: Get+Connected: Development and Pilot Testing of an Intervention to Improve Computer and Internet Attitudes and Internet Use Among Women Living With HIV
Source: JMIR Res Protoc. 2017 Mar 31;6(3):e50. doi: 10.2196/resprot.6391 (PMC5392213; doi:10.2196/resprot.6391)
Supplement: Multimedia Appendix 4 [file resprot_v6i3e_app4.ppt]

## Slide 1
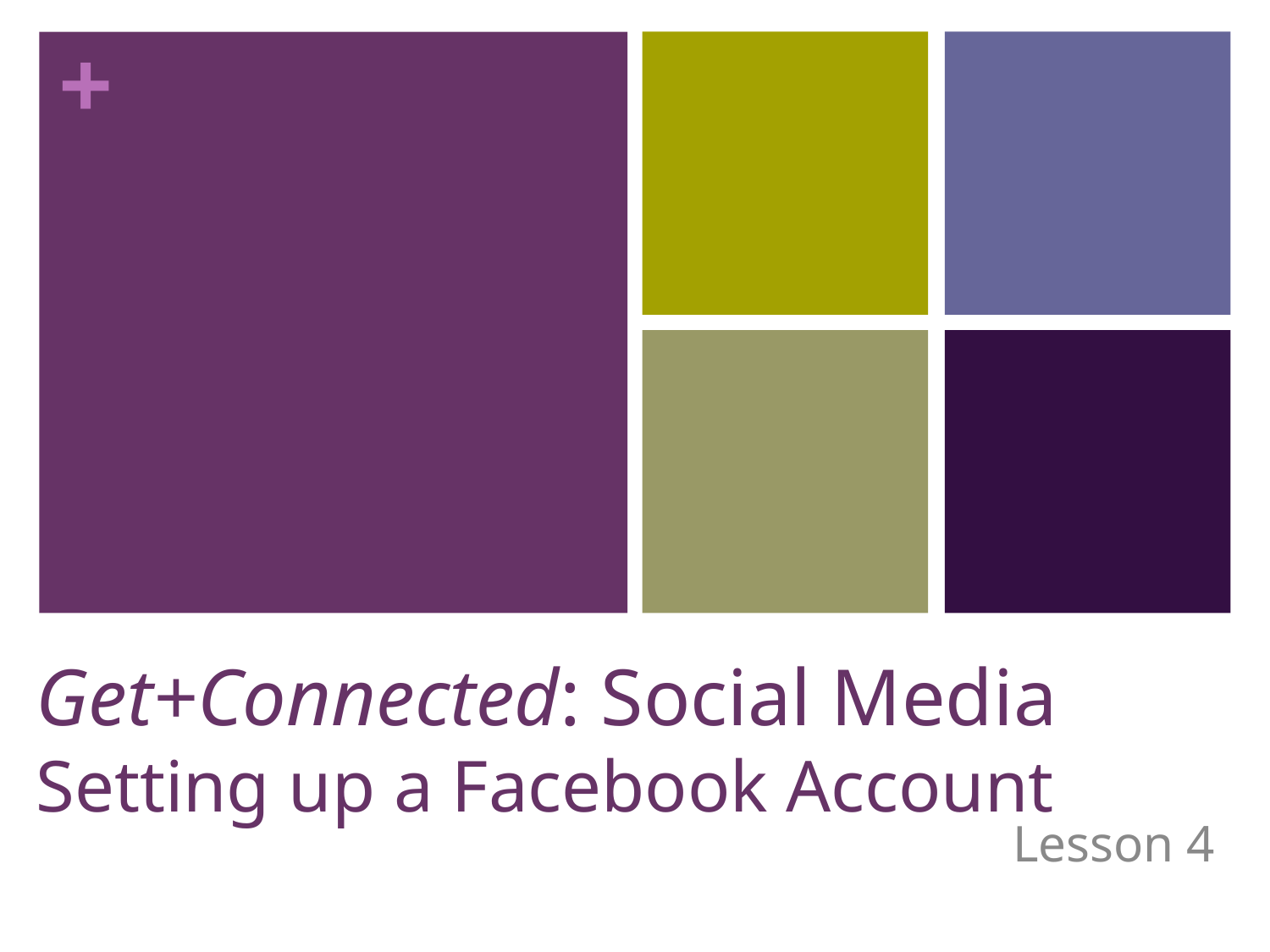

# Get+Connected: Social Media Setting up a Facebook Account
Lesson 4

## Slide 2
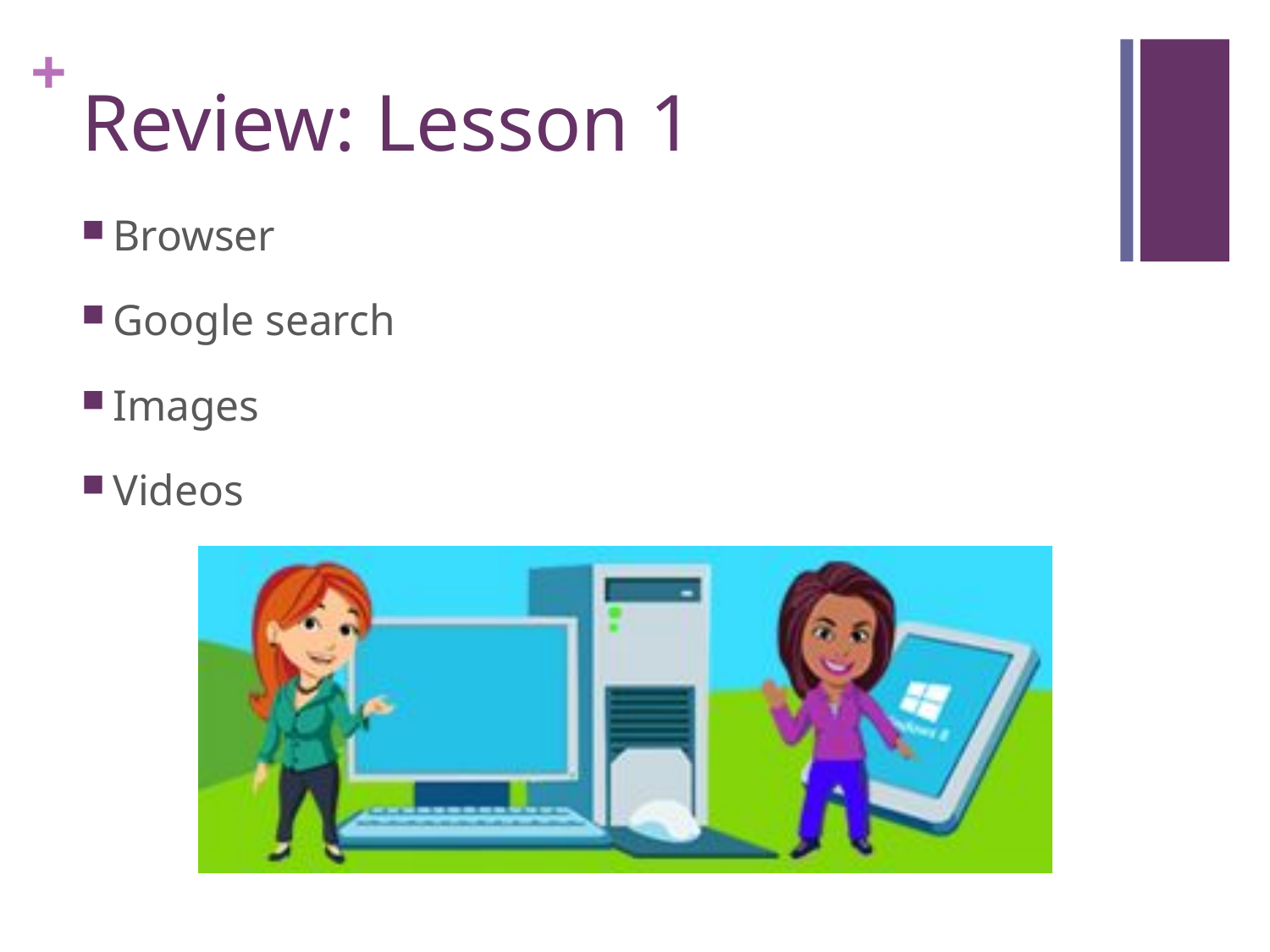

# Review: Lesson 1
Browser
Google search
Images
Videos

## Slide 3
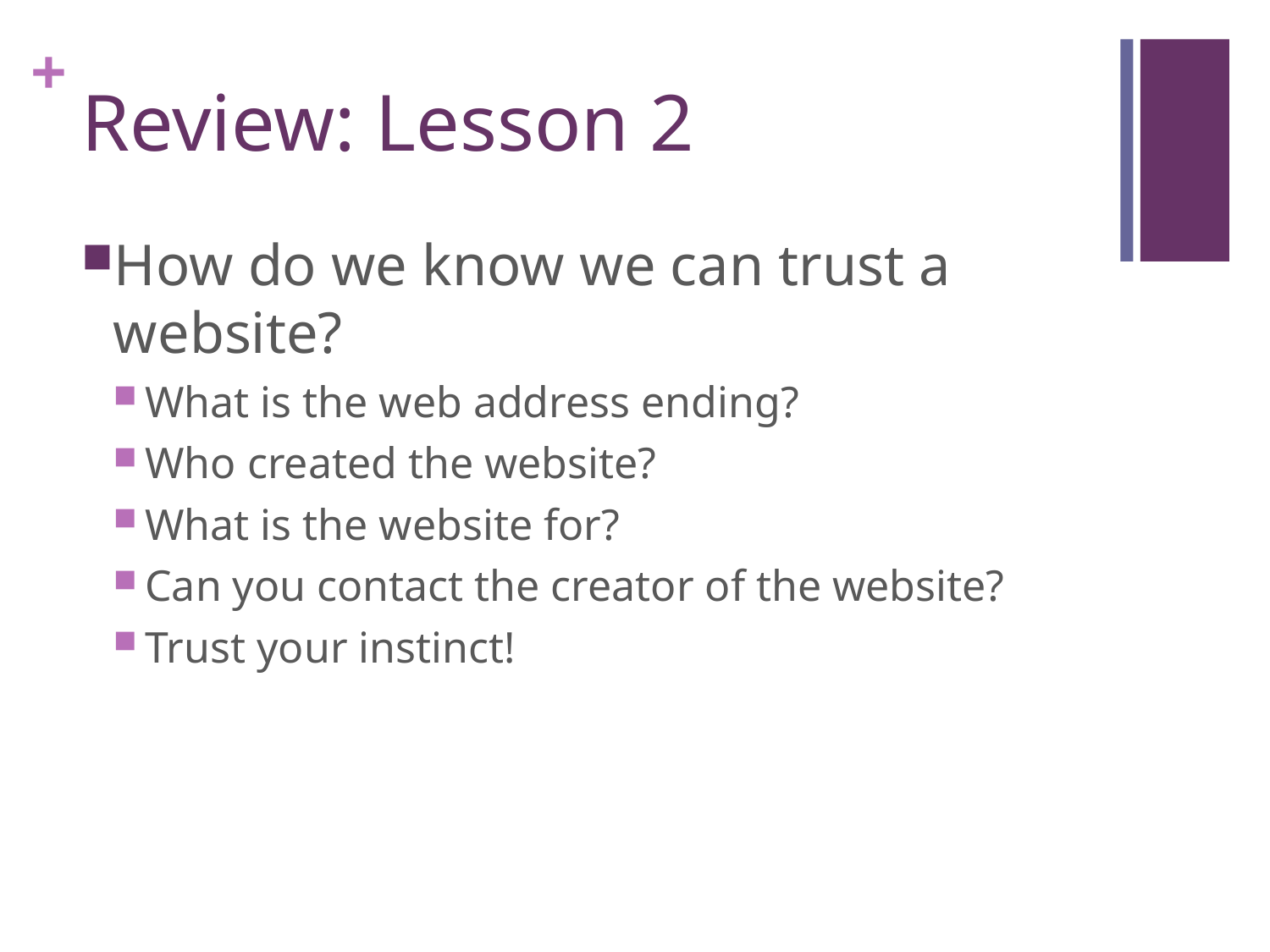

# Review: Lesson 2
How do we know we can trust a website?
What is the web address ending?
Who created the website?
What is the website for?
Can you contact the creator of the website?
Trust your instinct!

## Slide 4
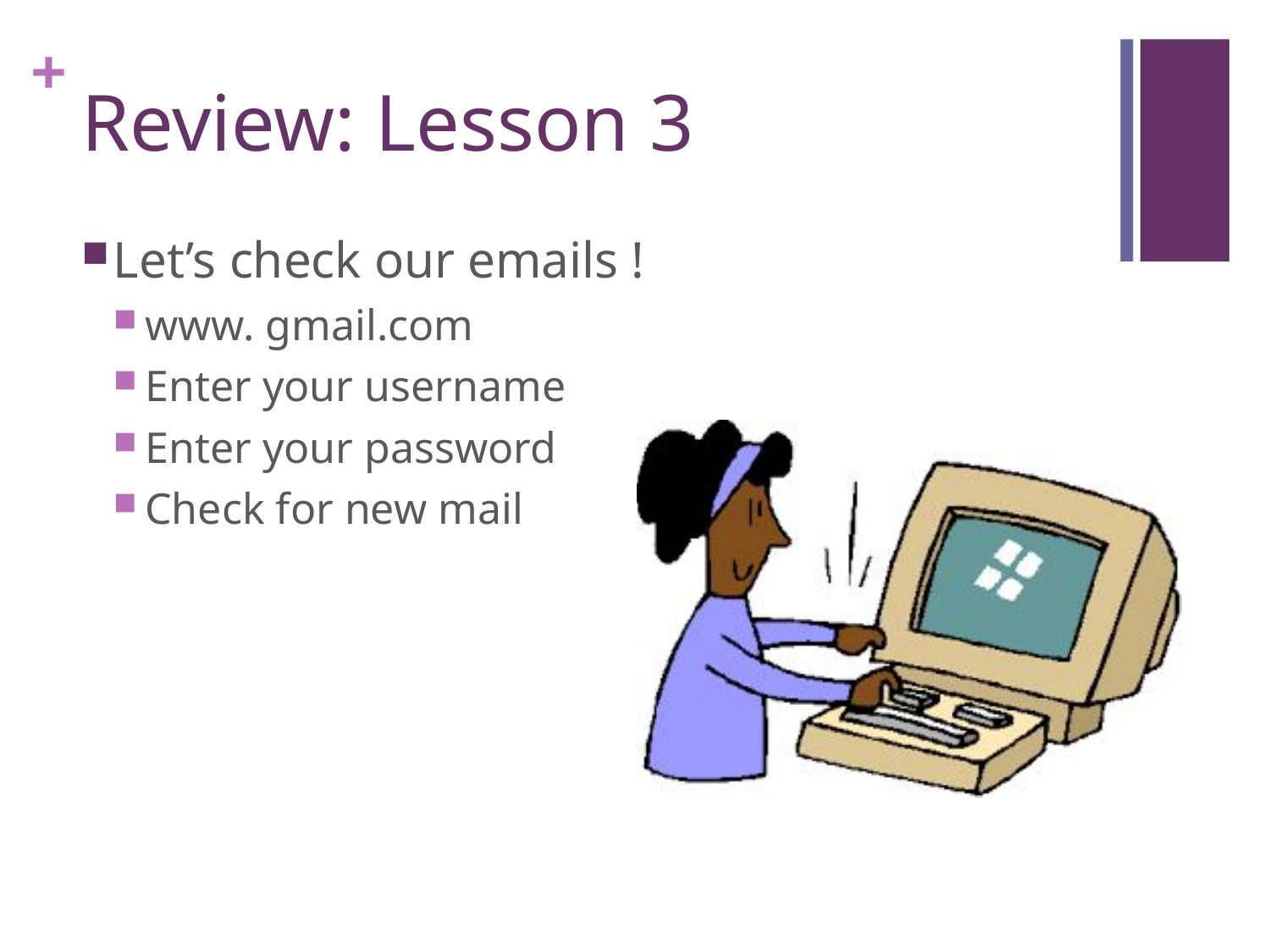

# Review: Lesson 3
Let’s check our emails !
www. gmail.com
Enter your username
Enter your password
Check for new mail

## Slide 5
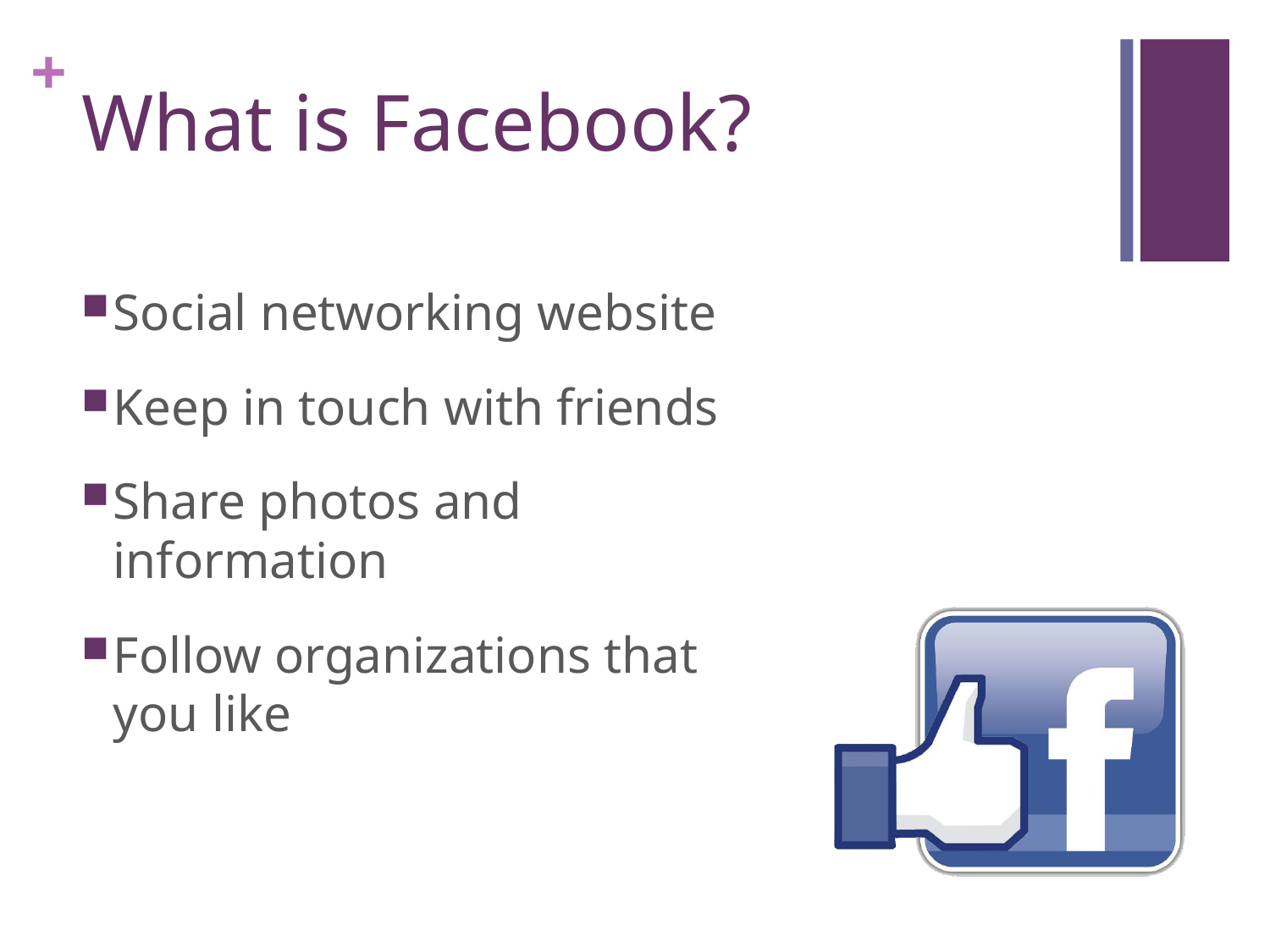

# What is Facebook?
Social networking website
Keep in touch with friends
Share photos and information
Follow organizations that you like

## Slide 6
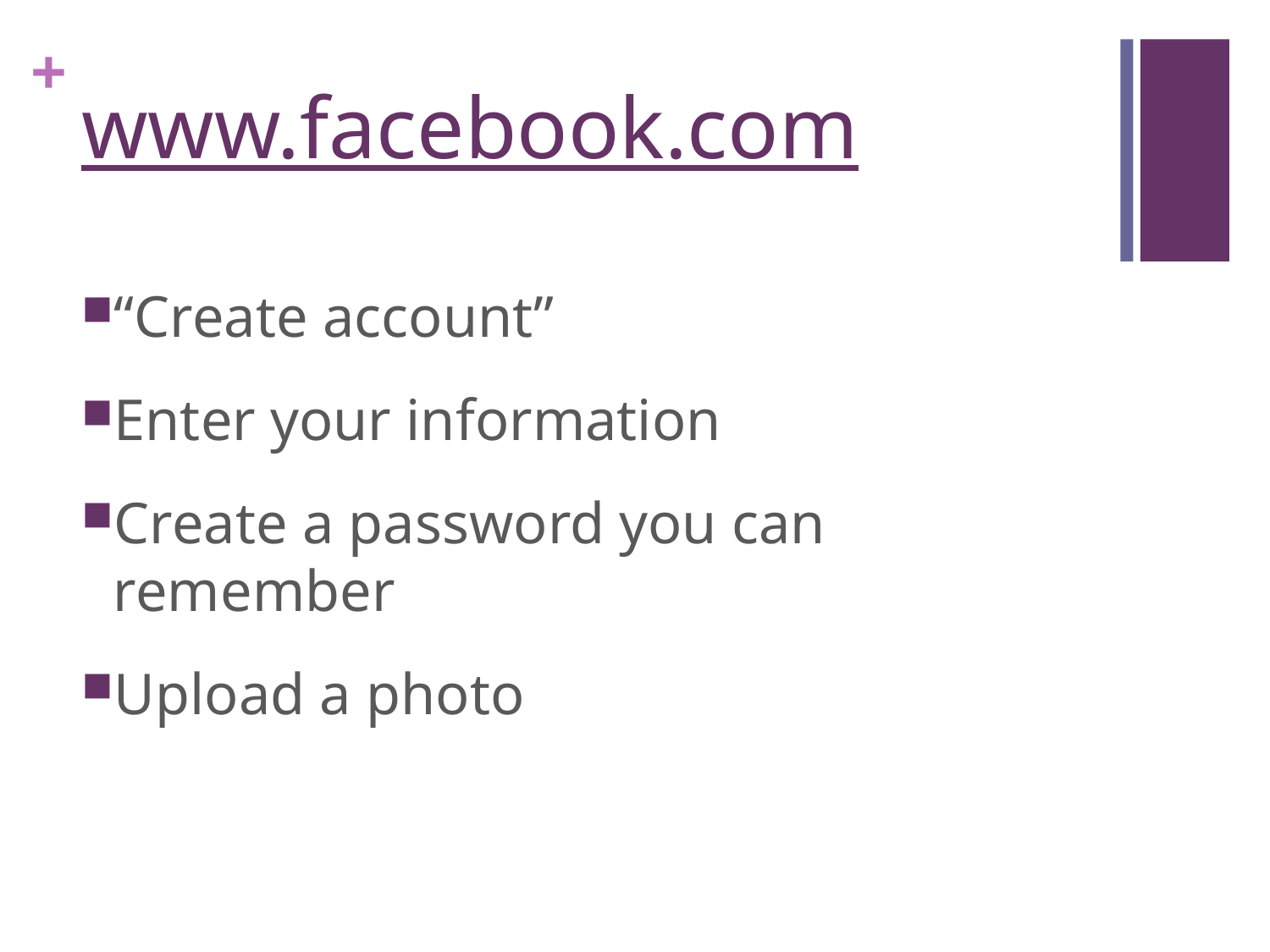

# www.facebook.com
“Create account”
Enter your information
Create a password you can remember
Upload a photo

## Slide 7
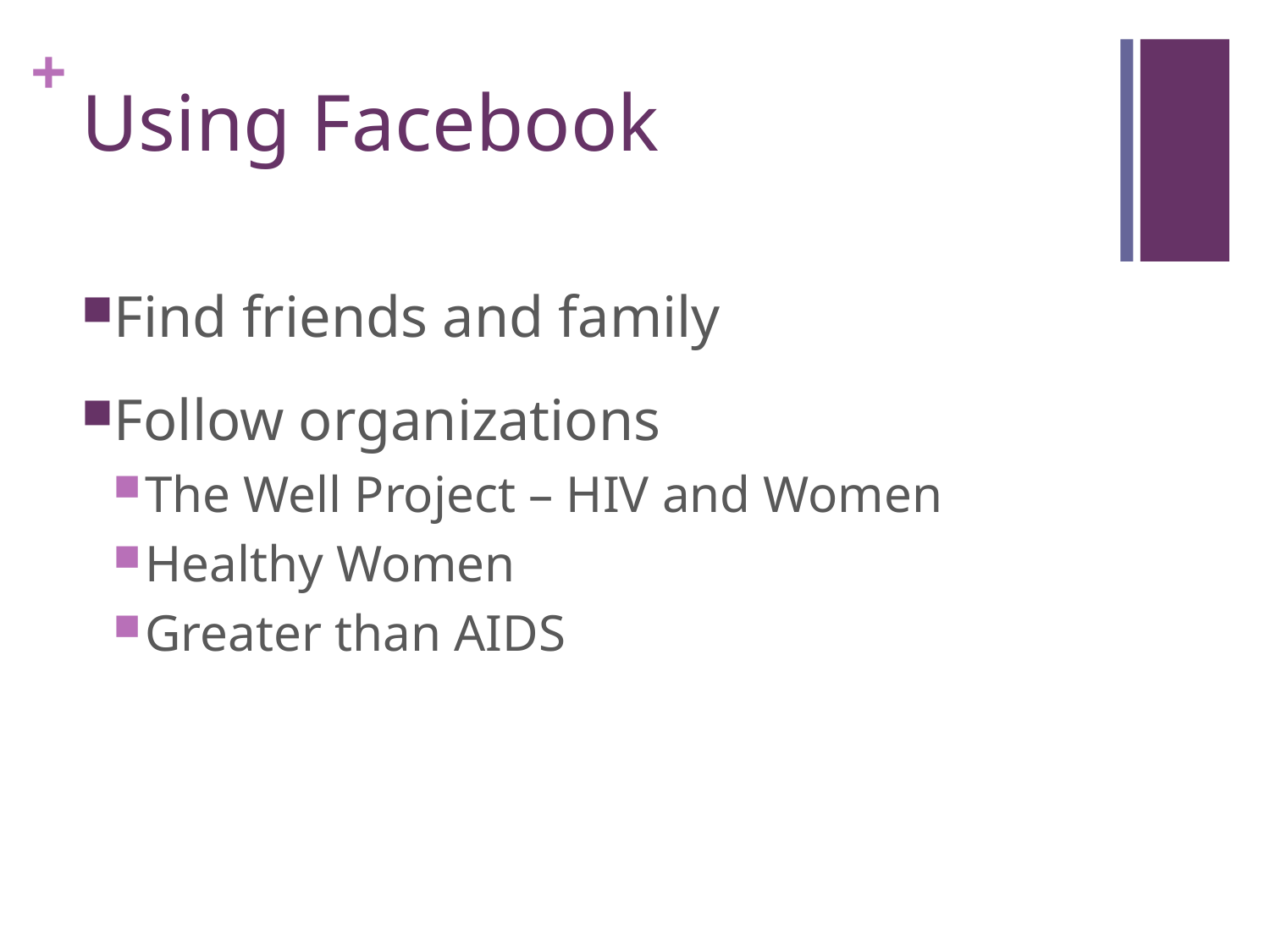

# Using Facebook
Find friends and family
Follow organizations
The Well Project – HIV and Women
Healthy Women
Greater than AIDS

## Slide 8
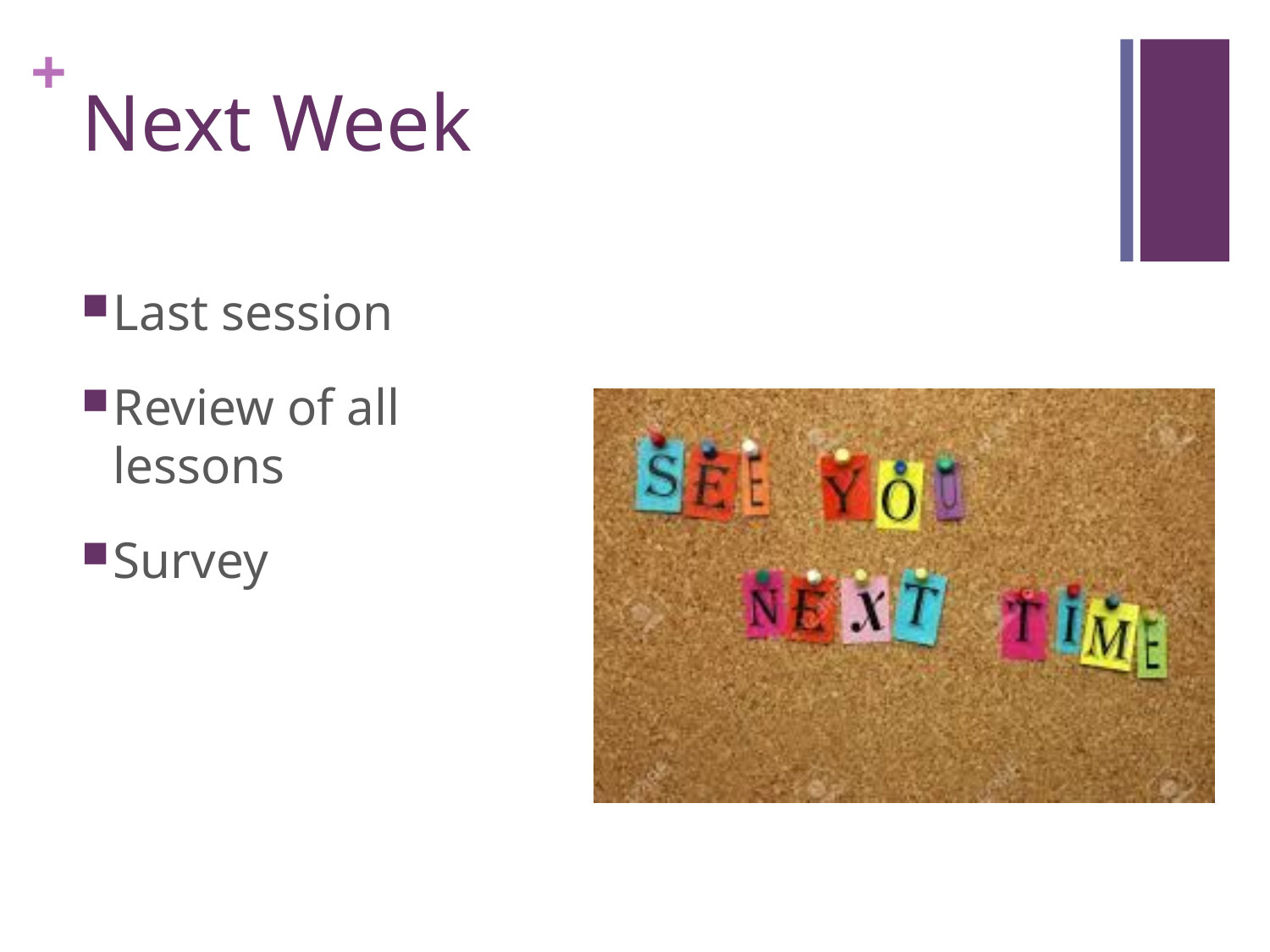

# Next Week
Last session
Review of all lessons
Survey
